# Mobile Tunnel Lining Measurable Image Scanning Assisted by Collimated Lasers

**DOI:** 10.3390/s25134177

**Published:** 2025-07-04

**Authors:** Xueqin Wu, Jian Ma, Jianfeng Wang, Hongxun Song, Jiyang Xu

**Affiliations:** School of Automobile, Chang’an University, Xi’an 710064, China; 2017022002@chd.edu.cn (X.W.); wjfchd@chd.edu.cn (J.W.); songhongx@chd.edu.cn (H.S.); 2017022009@chd.edu.cn (J.X.)

**Keywords:** road tunnel lining, measurement, laser, dual-quaternion, calibration

## Abstract

**Highlights:**

**What are the main findings?**

- A novel mobile tunnel lining scanning method aided by collimated lasers is presented, significantly improving image-stitching accuracy.
- A complete measurement system was built, and a Laplace kernel, maximum correntropy criterion, camera-pose calibration algorithm was introduced to further enhance calibration precision.

**What is the implication of the main finding?**

- The proposed approach yields near-seamless stitched images of tunnel linings.
- Using the new calibration algorithm, when outliers increase from 0% to 25%, the Euler-angle error grows by about 44%, and the translation error by roughly 45%, outperforming comparable benchmark algorithms.

**Abstract:**

The health of road tunnel linings directly impacts traffic safety and requires regular inspection. Appearance defects on tunnel linings can be measured through images scanned by cameras mounted on a car to avoid disrupting traffic. Existing tunnel lining mobile scanning methods often fail in image stitching due to the lack of corresponding feature points in the lining images, or require complex, time-consuming algorithms to eliminate stitching seams caused by the same issue. This paper proposes a mobile scanning method aided by collimated lasers, which uses lasers as corresponding points to assist with image stitching to address the problems. Additionally, the lasers serve as structured light, enabling the measurement of image projection relationships. An inspection car was developed based on this method for the experiment. To ensure operational flexibility, a single checkerboard was used to calibrate the system, including estimating the poses of lasers and cameras, and a Laplace kernel-based algorithm was developed to guarantee the calibration accuracy. Experiments show that the performance of this algorithm exceeds that of other benchmark algorithms, and the proposed method produces nearly seamless, measurable tunnel lining images, demonstrating its feasibility.

## 1. Introduction

The Road Tunnel Lining (RTL) is an essential component of road tunnels. It supports the surrounding rock to prevent collapse, inhibits groundwater infiltration, and enhances the tunnel’s appearance [1,2]. The technical condition of the tunnel lining directly affects the health of the tunnel and is closely related to traffic safety [3,4]. Owing to factors such as natural disasters, pressure [1,5,6], and groundwater erosion [7], RTLs inevitably develop defects such as cracking, leakage, spalling, and erosion. If these defects are not detected, evaluated, or repaired promptly, they may gradually worsen and threaten traffic safety. RTL inspection is a prerequisite for maintenance operations, requiring inspectors to work on-site with detection equipment. Inefficient inspection equipment means that inspectors must occupy the tunnel for extended periods, leading to traffic disruptions. Furthermore, low-precision equipment can result in distorted results, adversely affecting maintenance decisions.

The inspection of RTL mainly includes the detection of RTL deformation (RTL-D), RTL appearance defects (RTL-ADs), and RTL internal defects (RTL-IDs). RTL-D refers to the measurement of the deviation of the actual cross and longitudinal-sectional dimensions of the tunnel lining relative to the standard dimensions. RTL-ADs detection refers to the measurement of the distribution, length, width, and area of surface defects such as cracks and leakage on the lining. RTL-IDs detection refers to the detection of internal defects such as voids within the lining. In practice, inspectors typically perform a preliminary evaluation of the tunnel based on RTL-ADs detection results and then decide whether to conduct more resource-intensive RTL-D or RTL-IDs detections. Therefore, improving the efficiency of RTL-ADs detection is crucial for improving operational efficiency. RTL-ADs can be measured by capturing images with cameras and performing analysis.

Equipped with onboard cameras, a Road Tunnel Lining Inspection Car (RIC) captures continuous, high-resolution, measurable panoramic images of the tunnel lining while driving through the tunnel without stopping. The captured RTL images allow inspectors to assess the tunnel condition remotely from an office [8]. Although the RIC has significantly improved field inspection efficiency, there is still room for improvement to further enhance efficiency and reduce costs. In recent years, many researchers have exploited deep learning techniques to perform automatic RTL-AD recognition on RIC image data, achieving high detection accuracies [9,10,11]. Enhancing the scanning precision of the RIC and lowering the overall hardware cost would complete this technology chain, substantially improving the efficiency and scalability of tunnel inspection and maintenance operations.

Owing to the large size of the tunnel lining, a single camera cannot cover the entire cross-section of the lining while maintaining high image resolution. As a result, current RICs rely on camera arrays for inspection. With camera arrays in use, the RIC’s data processing system must employ image stitching techniques to merge multiple images with overlapping areas into a single, complete image [12]. The image stitching process consists of the following three main stages: image registration, image reprojection, and image blending.

Key challenges for tunnel defect detection arise during the image registration stage. The goal of image registration is to estimate the geometric relationship between two images with overlapping areas, using models such as translation, affine transformation, or perspective transformation. Standard image registration techniques typically rely on extracting the corresponding features from overlapping regions to estimate the model parameters [13]. However, these methods often fail in scenarios such as tunnel linings, where few features are available for registration [14], especially when the overlapping fields of view (FOVs) between cameras are small.

In this paper, an RTL scanning method based on a collimated laser array is proposed to improve the stitching accuracy of lining images. A RIC based on the proposed method was developed, and a Laplace kernel, maximum correntropy criterion (MCC)-based algorithm is developed to enhance the calibration accuracy of the system. The experimental results demonstrate that the algorithm can accurately estimate the pose of adjacent cameras. With the assistance of collimated laser spots, the system can generate near-seamless, high-quality RTL panoramic images.

The remainder of this paper is organized as follows: Section 2 introduces related research. Section 3 presents the schematic of the developed system. Section 4 describes the methodology. Section 5 presents experiments and discussions. Finally, Section 6 provides the conclusions.

## 2. Background

Because the FOV of a single camera is insufficient to encompass an entire tunnel lining cross-section at the resolution required for metric analysis, state-of-the-art RICs employ camera arrays. The fundamental technical problems in mobile RTL imaging are as follows:

(1) Image reprojection—establishing accurate mapping between each camera’s image and the three-dimensional lining surface. (2) Image registration—fusing the individual views into a seamless, metrically consistent panoramic image of the full lining.

Existing solutions can be classified into the following three methodological families: (1) Pure photogrammetric stitching, which relies solely on inter-image features; (2) LiDAR-assisted stitching, which incorporates point cloud geometry as an external constraint; (3) Collimated laser-assisted stitching, which exploits structured-light references to refine both projection and alignment. Table 1 lists the principal sensors adopted in commercial and research-grade RICs worldwide. As the table shows, most systems implement either the pure-photogrammetric or the LiDAR-assisted strategy, whereas only a few employ the more recent collimated laser approach.

Pure-photogrammetric stitching estimates the image-to-lining projection for each camera by combining the pre-calibrated camera extrinsics with an approximate camera-to-lining distance. Subsequently, adjacent camera images are registered with a simple similarity (translation) operator and concatenated. References [28,29] describe an RTL image stitching method based on the corresponding region matching. This method first computes the translation between images based on the similarity of overlapping regions, stitches the images into long-strip images, and finally assembles the long-strip images into a complete panoramic image of the lining. The method, however, is highly sensitive to vehicle pitch, bounce, or yaw; to minimize such disturbances the vehicle must travel at low speed, which severely degrades inspection efficiency. Pahwa et al. [33,34] introduced a cylindrical-tunnel image stitching strategy in which an initial mosaic is generated from the known tunnel cross-section to produce a coarse projection, after which a bundle adjustment refines the alignment using feature points. Their prototype system demonstrated that panoramic stitching is achievable, yet its robustness still depends on the quality of feature extraction. Jiang et al. [35] developed a RTL scanning system based on LSCs. Common features in the overlap of two adjacent LSC images are matched to accomplish lateral stitching, while the native LSC acquisition sequence provides longitudinal concatenation automatically, noticeably simplifying the pipeline. Nevertheless, the approach presupposes a feature-rich lining surface; in low-texture areas—scenarios frequently encountered when the camera FOV is narrow—reliable corresponding points cannot be obtained, causing the fine registration to fail.

LiDAR-assisted stitching adopts a coarse-to-fine strategy for image stitching [17,36,37]. First, rough stitching is performed based on the calibration pose between the cameras and the LiDAR. The corresponding feature-based image registration techniques are then used for fine stitching. Wang et al. [14] use LiDAR measurements of the camera-to-lining pose for coarse alignment and refine the mosaic with SURF feature points extracted from the overlapping regions. Their experiments report a SURF success rate of only about 66%, reflecting the general paucity of salient features in tunnel lining imagery. Du et al. [16] propose a “seam-driven” strategy. LiDAR again supplies the coarse match, whilst the final panorama is generated by optimizing the stitching seams with a graph cut blender, thus avoiding strict point-to-point registration. Although robust, the graph cut is computationally expensive—0.52 s per image for coarse alignment versus 42.7 s per image for seam optimization. Zou et al. [31] calibrate LSC-to-LiDAR extrinsics via a pyramidal calibration block and a large striped board. The intrinsic projection is first used for coarse alignment, a Bayesian scheme then increases pairwise registration accuracy in low-texture or corroded regions, and a graph cut refines the seam. Nevertheless, the entire pipeline remains computationally intensive. Overall, LiDAR assistance delivers substantially better accuracy than pure-photogrammetric stitching methods but it still incurs high computational cost for feature/region matching, and, in feature-deficient scenes, the fine stage may fail, leading to visible seams.

Collimated laser-assisted stitching projects a laser array into the overlapping FOVs of adjacent cameras, creating artificial tie points that guarantee reliable correspondences even when the lining offers little native texture. Some researchers have explored the use of collimated lasers to assist with stitching ASC RTL images in a static environment. Wang et al. [38,39] proposed a collimated laser-assisted method that utilizes a laser array to project these spots as auxiliary control points, solving the P4P problem to establish the pose relationship between the lining surface and camera, thus facilitating image stitching, which demonstrated the feasibility of this approach, showing that the laser array provides sufficient constraints for accurate alignment. However, their experiments were limited to stationary setups and have not yet been extended to mobile tunnel lining inspection, where vehicle motion, vibration, and exposure timing introduce additional challenges. The commercial system FOCUSα-T [30] likewise employs a collimated laser array and publishes impressive panoramic results, but no technical details of its calibration or stitching algorithms have been disclosed.

**Figure 1:**
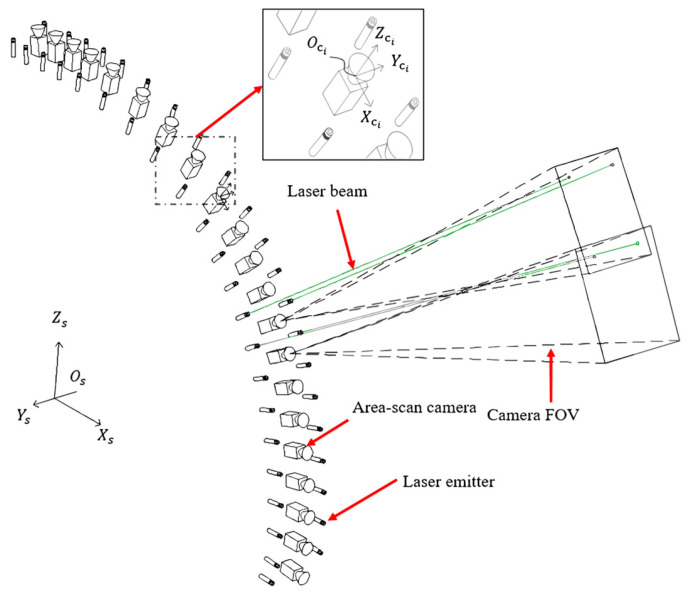
Schematic diagram of the working principle of the RTL measurement system (using the side lining measurement system for illustration). $O_{s}-X_{s}Y_{s}Z_{s}$ represents the coordinate system of the measurement system and $O_{ci}-X_{ci}Y_{ci}Z_{ci}$ represents the coordinate frame of the i-th camera.

The accurate calibration of the structural parameters of the measurement system is essential for the development of such equipment, including the poses of the cameras and lasers. The intrinsic parameters of a single camera and the poses of the lasers in a camera coordinate frame can be calibrated using Zhang’s method with a checkerboard [40]. However, for the tunnel lining scanning systems shown in Figure 1 the overlapping FOVs between cameras are limited, presenting challenges for the pose calibration between cameras. Furthermore, some of the cameras and lasers face to the sky in the system, making it necessary to suspend the calibration boards or instruments in the air for calibration. Therefore, to reduce the operational difficulty, the calibration device should be sufficiently lightweight and compact.

A substantial body of work has been devoted to calibrating camera arrays whose FOVs are small or entirely non-overlapping. Li et al. [41] employ a dual-ring circular-coded target, and by translating this large marker to multiple locations and exploiting the uniqueness of each coded dot they solve each camera’s pose with respect to the board and, in turn, recover inter-camera extrinsics. Although the method is accurate, the target itself is bulky and difficult to deploy in constrained spaces such as tunnels. Crombrugge et al. [42] replace the physical board with a projector that casts an encoded stripe pattern onto a planar screen; the approach inherits the same drawback—namely, the requirement for a large projection surface—rendering it equally unsuitable for tunnel lining scanners. Yang et al. [43,44] mount two chessboards on a rigid bar and apply Zhang’s technique to determine the relative pose, yet the rigid target is heavy and unwieldy in confined environments. Liu et al. [45] adopt a lightweight alternative in which a collimated laser beam is intercepted by a planar plate, and each camera observes the resulting spot to derive control points. While the procedure is operationally convenient, its accuracy is ultimately constrained by the precision of the laser rangefinder.

To address these limitations we propose a single-checkerboard, line feature-based calibration framework tailored to mobile tunnel lining scanners, in that the considered line features can provide more stable and reliable characteristics. The collimated laser is modeled with dual quaternions (DQ) [46,47,48], providing a compact algebraic representation that accelerates pose computation and improves numerical stability. Conventional DQ solvers rely on least squares optimization [49] and are therefore vulnerable to outliers arising from specular reflections or defocused images of the checkboard. Inspired by the method in reference [50], a Laplace kernel-based algorithm is developed to improve the pose estimation accuracy of cameras.

## 3. Schematic of the System

A schematic of the proposed method is shown in Figure 1, which integrates an ASC array with a collimated laser array to capture tunnel lining images. Two laser beams were projected onto the overlapping FOVs of adjacent cameras. These lasers, along with ASCs, form a triangulation measurement unit, allowing for the simultaneous acquisition of tunnel image and image projection relationship measurements. The projected laser spots provide auxiliary reference points for stitching the lining images, thereby addressing the problem of image registration difficulties owing to the lack of features.

**Figure 2:**
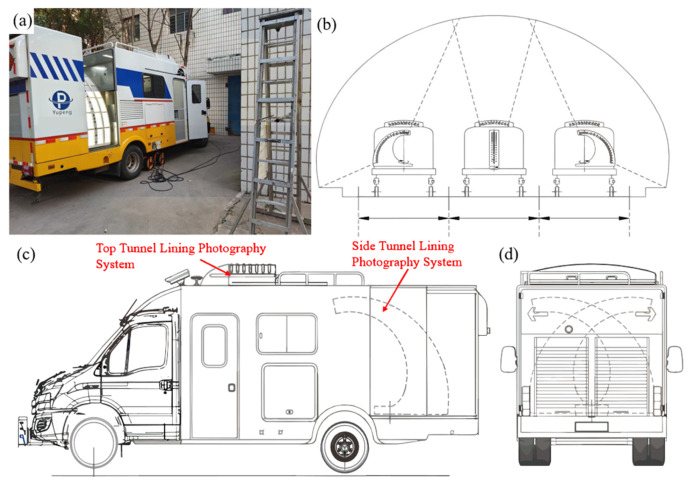
External views of the tunnel lining measurement system. (**a**) Overall appearance of the measurement system; (**b**) schematic diagram of the measurement system’s operation process; (**c**) side view of the measurement system; (**d**) rear view of the measurement system.

An RIC was developed based on this method, as illustrated in Figure 2a. The system comprised a top RTL photogrammetry setup mounted on the roof of the vehicle and a rotatable setup mounted at the rear (see Figure 2c). LED strobe lights served as the light source for image capture. As shown in Figure 2b, the top-mounted system is used to inspect large-span tunnel linings (with three or more lanes), whereas the rotatable rear system measures the side linings. The top system consisted of 11 narrow-FOV ASCs and 1 wide-FOV ASC, and the side system contained 21 narrow-FOV ASCs and 3 wide-FOV ASCs. All cameras are Basler acA2440-75 um/uc sensors (2440 × 2048 px; mono/color camera; Basler AG, Ahrensburg, Germany) fitted with C-mount fixed-focus lenses. The narrow-FOV cameras capture fine surface texture, whereas the wide-FOV cameras provide macroscopic context. The system includes 68 collimated lasers, with 2 lasers shared between adjacent cameras. Each ASC, together with the four lasers, forms a triangulation measurement unit. To prevent defocus, the system is equipped with a focus-adjustment mechanism for each camera lens, driven by a stepper motor, to ensure sharp images of the linings in the varied tunnel shapes.

For highway operation the inspection vehicle must maintain a cruising speed of 60 to 80 km/h. To suppress the motion-induced smear generated during exposure, the exposure time of each ASC is restricted to ≤10 µs. Because tunnel linings are generally gray-to-black and ambient lighting is extremely weak, high-intensity artificial illumination is mandatory. The developed lighting system adopts a modular LED strobe design. The side tunnel lining photography system is equipped with 160 LED modules, and the top one is assembled with 120 LED modules: each module contains 18 LED chips rated at 18 W. The collimated laser emitters deliver 70 mW at 520 nm. Given the large aggregate power of the lasers and LEDs, continuous operation would generate excessive heat, risking hardware damage, accelerating optical degradation, and increasing energy consumption.

**Figure 3:**
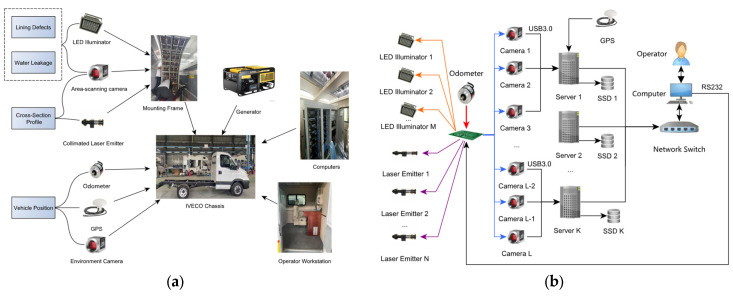
System architecture. (**a**) Key assemblies; (**b**) sensor-data acquisition framework.

The hardware architecture, as shown in Figure 3a, is proposed to mitigate these issues, with a pulse-distribution controller centrally scheduling the on–off timing of the LEDs, lasers, and cameras. Both lasers and LEDs are fired slightly before the start of camera exposure to ensure maximum irradiance during the exposure window. Each laser pulse lasts 1 ms, each LED pulse 40 µs, and the maximum repetition rate is about 72 Hz.

As shown in Figure 3b, every server computer controls 3 ASCs. Each server (Model AIIS-3410U, Advantech Co., Suzhou, China; Intel i7-6700 CPU, 8 GB RAM, Intel, Santa Clara, CA, USA) runs an independent acquisition service, which is orchestrated by a supervisory workstation. The data pipeline of the acquisition service is implemented in C++; message queues connect independent threads for each functional stage, converting raw image streams to JPEG streams and writing them to solid-state drives (SSDs).

The models, key specifications, and quantities of all major sensor components are summarized in Table 2.

## 4. Methodology

### 4.1. RTL Scanning Schematic

**Figure 4:**
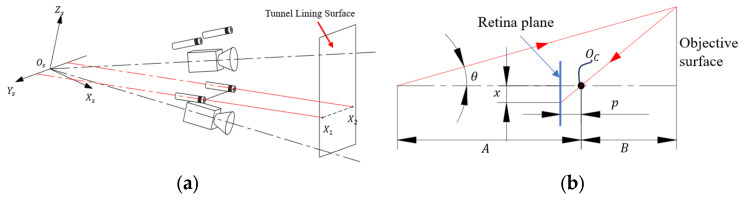
Ideal measurement model. (**a**) Schematic diagram of the system. (**b**) Side view of the system. $O_{s}-X_{s}Y_{s}Z_{s}$ is the coordinate frame of the measurement system, $X_{1}$ and $X_{2}$ are the coordinates of the laser spots in object space, x is the longitudinal coordinate of the spot in retina plane, $O_{c}$ is the optical center of the camera, θ it the angle between the collimation laser and the camera’s optical axis, p it the principle distance of the lens, A is the baseline distance of the system, and B is the distance from $O_{c}$ to the objective surface.

The principle of collimation laser-assisted photogrammetry is illustrated in Figure 4b. The optical axes of the cameras converged at the origin $O_{s}$ of the measurement system, and the collimation laser beams are parallel and intersected with the $Y_{s}$ axis of the coordinate system. According to the principles of triangular geometry, the relative distance of the laser spots in the object space from the camera is determined by the following relationship:

(1) $$B=\frac{Ap\tan\theta }{x-p\tan\theta }\;\;x>p\tan\theta$$

Equation (1) can be used to design the structural parameters of the system. In practice, the laser spot position is given by the following:

(2) $$z_{c}\tilde{u}=z_{c}\left[\begin{array}{c} u\\ v\\ 1 \end{array}\right]=\left[\begin{array}{ccc} k_{x} & 0 & u_{0}\\ 0 & k_{y} & v_{0}\\ 0 & 0 & 1 \end{array}\right]\left[\begin{array}{c} x_{c}\\ y_{c}\\ z_{c} \end{array}\right]=KX^{\left(c\right)}$$

where $k_{x}=p/d_{x}$ $k_{y}=p/d_{y}$, p is the principal distance of the lens, $d_{x}$ and $d_{y}$ are the pixel dimensions of the camera sensor, K is the camera intrinsic matrix, and the superscript on $X^{\left(c\right)}$ is the point coordinate in the camera coordinate system (c-frame). u and v are the coordinates of point X in the image and $u_{0}$ and $v_{0}$ are the projection coordinates of the optical axis and the intersection of the imaging plane in the image.

Before using Equation (2), the lens distortion values must be calculated and corrected. The lens distortion is described using the second-order Brown model, expressed as follows:

(3a) $$x_{1cd}=x_{c1}\left(1+k_{1}r^{2}+k_{2}r^{4}\right)$$

(3b) $$y_{1cd}=y_{c1}\left(1+k_{1}r^{2}+k_{2}r^{4}\right)$$

where $k_{1}$ and $k_{2}$ are the radial distortion coefficients, $x_{c1}$ and $y_{c1}$ are the undistorted normalized image plane coordinates, $x_{1cd}$ and $y_{1cd}$ are the distorted normalized image plane coordinates, and $r^{2}=x_{c1}^{2}+y_{c1}^{2}$.

According to Equation (2), once the collimated laser position is given the three-dimensional position of the laser spot in the c-frame can be computed. Let the Plücker coordinate of the laser beam by the authors of [47] be as follows:

(4) $$L={\left[\begin{array}{cc} L^{T} & L_{O}^{T} \end{array}\right]}^{T}$$

where L and $L_{O}$ are both 3×1 matrices, L is the direction vector of the line, $L_{O}$ is the moment of the line, and $L^{T}L_{O}=0$. Given two points $X_{1}$ and $X_{2}$ on the three-dimensional line, the Plücker coordinates are given by the following:

(5a) $$L=X_{2}-X_{1}$$

(5b) $$L_{O}={\left[X_{1}\right]}_{\times }X_{2}$$

where ${\left[\cdot \right]}_{\times }$ is the following skew-symmetric matrix operator:

(6) $${\left[X\right]}_{\times }=\left[\begin{array}{ccc} 0 & -X_{3} & X_{2}\\ X_{3} & 0 & -X_{1}\\ -X_{2} & X_{1} & 0 \end{array}\right]$$

When the laser line direction is normalized, i.e., $\left\|L\right\|=1$, the coordinates of any point on the collimated laser beam can be written as follows:

(7) $$X=\lambda L+{\left[L\right]}_{\times }L_{O}$$

where λ∈R is an arbitrary scalar.

Substituting Equation (7) into Equation (2) yields the following laser spot localization equation:

(8) $$K^{-1}\tilde{u}=\lambda L+\eta {\left[L\right]}_{\times }L_{O}$$

where λ, η∈R are unknown scalars. Equation (8) can be solved via Singular Value Decomposition (SVD), thereby recovering the three-dimensional position of the laser spot in the object space.

### 4.2. Reprojection and Stitching of Tunnel Lining Images

The tunnel lining profile can be treated as a curve consisting of multiple arc segments. The tunnel lining surface within the FOV of a camera can be approximated as a plane owing to the small curvature of the tunnel profile and limited FOV of a single camera. Based on this assumption, the projection relationship between the image and the tunnel lining surface can be determined using the coordinates of the laser spots in the image.

As shown in Figure 4a, there are 4 laser spots in the FOV of each camera, which can be used to determine the projection plane. For readability, the superscripts of the coordinate frames are temporarily omitted. According to Equation (8), the object-side coordinates of the 4 laser spots, ${\tilde{X}}_{1}$, ${\tilde{X}}_{2}$, ${\tilde{X}}_{3}$, ${\tilde{X}}_{4}$, are obtained, and these points are coplanar according to the assumption. The normal of this plane is $\varepsilon ={\left[\begin{array}{ccc} \varepsilon_{x} & \varepsilon_{y} & \varepsilon_{z} \end{array}\right]}^{T}$, and the following relation holds:

(9) $$\begin{array}{c} {\left[\begin{array}{cccc} {\tilde{X}}_{1} & {\tilde{X}}_{2} & {\tilde{X}}_{3} & {\tilde{X}}_{4} \end{array}\right]}^{T}\varpi =0_{4} \end{array}$$

where $\varpi ={\left[\begin{array}{cc} \varepsilon^{T} & \xi \end{array}\right]}^{T}$ is the projection plane.

Solving Equation (9) with SVD, the projection relationship between the tunnel lining surface and the camera image is given by the following:

(10) $$z_{c}\left[\begin{array}{c} \tilde{u}\\ 0 \end{array}\right]=\left[\begin{array}{c} \left[\begin{array}{cc} K & 0 \end{array}\right]\\ \varpi \end{array}\right]{\tilde{X}}^{\left(c\right)}=\left[\begin{array}{cc} K & 0\\ \eta^{T} & \xi \end{array}\right]{\tilde{X}}^{\left(c\right)}$$

where ξ≠0 to ensure that M is invertible. This formula can be used to calculate the coordinates in c-frame from $\tilde{u}$ through the following:

(11) $$X^{\left(c\right)}=-\frac{K^{-1}\tilde{u}}{\xi^{-1}\eta^{T}K^{-1}\tilde{u}}$$

The pixel-scale normalized RTL image can be obtained using Equations (10) and (11). The steps are as follows:

(1) Equation (10) was used to project the border pixels of the image to determine the boundaries of the new image;
(2) Equation (11) was used to calculate the backward interpolation mapping table of the new image, and interpolation is then performed to generate the new image.

**Figure 5:**
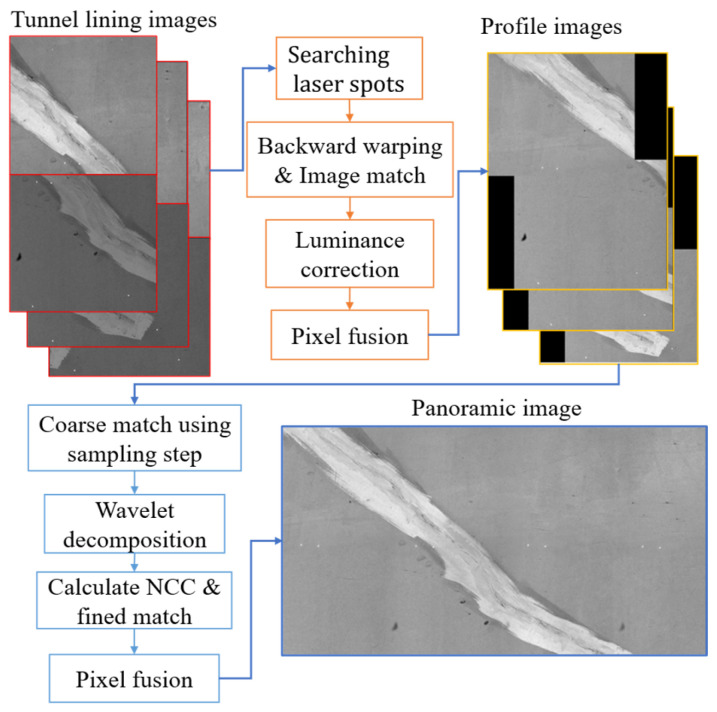
Flowchart of tunnel lining image stitching.

The pixel-scale normalized RTL images must be stitched. The following stitching process is adopted (as shown in Figure 5):

(1) The two-dimensional affine transformation parameters between two adjacent camera images are calculated based on the corresponding laser spots. Using these parameters, backward warping was applied to the benchmark camera images according to Equation (12). Obtain the overlapping region of the images, adjust the grayscale of the images, and finally perform pixel fusion within the overlapping region to generate the RTL profile images.
(2) The overlapping region of the adjacent RTL profile images is roughly calculated based on the camera acquisition interval. Wavelet decomposition is then performed on the images in this region to separate the high- and low-frequency images. Next, Equation (13) is used to calculate the normalized cross-correlation (NCC) of the overlapping region between the two high-frequency images and to find its maximum position to achieve precise registration of adjacent profile images. Finally, the following pixel fusion was performed in the overlapping region to obtain a panoramic RTL image:

(12) $$u_{2}=Hu_{1}+h$$

(13) $$NCC\left(I_{1},I_{2}\right)=\frac{\sum_{u,v}\left[I_{1}\left(u,v\right)-{\bar{I}}_{1}\right]\left[I_{2}\left(u,v\right)-{\bar{I}}_{2}\right]}{{\left(\sum_{u,v}{\left[I_{1}\left(u,v\right)-{\bar{I}}_{1}\right]}^{2}\sum_{u,v}{\left[I_{2}\left(u,v\right)-{\bar{I}}_{2}\right]}^{2}\right)}^{1/2}}$$

where H is the affine transformation matrix, s is the scaling parameter, θ is the rotation angle, h is the two-dimensional translation vector, $I_{1}$ and $I_{2}$ are the pixel grayscale values of the two images, and

(14) $$H=s\left[\begin{array}{cc} 1 & \theta \\ -\theta & 1 \end{array}\right]$$

### 4.3. Fast Search for Laser Spot in Image

Before image projection, it is necessary to obtain the image coordinates of the laser spots. Because the laser spot occupies a very small proportion of the camera image, applying a global image search algorithm would result in inefficient system operation. By utilizing the projection constraint of the laser beams and the epipolar constraint between the cameras, the laser spot can be found quickly.

Let the coordinates of the corresponding point X in the two camera coordinate frames be $X^{\left(c_{1}\right)}$ and $X^{\left(c_{2}\right)}$, then the following can be obtained:

(15) $$X^{\left(c_{2}\right)}=RX^{\left(c_{1}\right)}+t$$

The epipolar constraint is described as follows [51]:

(16) $${\tilde{u}}_{1}^{T}F{\tilde{u}}_{2}=0$$

where $F=K_{2}^{-T}EK_{1}^{-1}$ is the fundamental matrix between the cameras and $E={\left[t\right]}_{\times }R$ is the essential matrix.

Let the Plücker coordinates of the laser beam in the $c_{1}$-Frame be $L^{\left(c_{1}\right)}$, then its projection in the image of camera 2 is as follows [52]:

(17) $$l_{2}\sim K_{2}^{-T}\left[\begin{array}{cc} {\left[t\right]}_{\times }R & R \end{array}\right]L^{\left(c_{1}\right)}$$

According to Equation (17), the projection of $L^{\left(c_{1}\right)}$ in the image coordinate frame of camera 1 is as follows:

(18) $$l_{1}\sim K_{1}^{-T}\left[\begin{array}{cc} 0_{3\times 3} & I_{3} \end{array}\right]L^{\left(c_{1}\right)}=K_{1}^{-T}L_{O}$$

**Figure 6:**
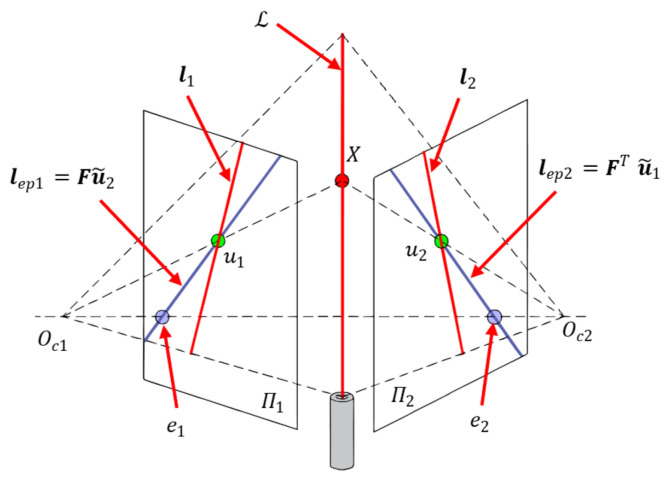
Fast search for laser spot based on imaging geomatic constraints. L is the Plücker coordinate of the laser, X is a point on L, $\Pi_{1}$ and $\Pi_{2}$ are the image planes of cameras $c_{1}$ and $c_{2}$, $u_{1}$ and $u_{2}$ are the projections of X on $\Pi_{1}$ and $\Pi_{2}$, $l_{1}$ and $l_{2}$ are the projections of L on $\Pi_{1}$ and $\Pi_{2}$, $e_{1}$ and $e_{2}$ are the epipoles, and $l_{ep1}$ and $l_{ep2}$ are the epipolar lines.

Using Equations (16)–(18), a set of constraint lines for the corresponding point X can be obtained, as shown in Figure 6. Based on these lines, the following coarse-to-fine strategy can be used to quickly locate $u_{1}$ and $u_{2}$:

(1) Search along the line $l_{1}$ to find ${\tilde{u}}_{1}$, and then calculate ${\tilde{u}}_{2,rough}=l_{ep2}\times l_{2}$ for a coarse location of $u_{2}$;
(2) In the vicinity of ${\tilde{u}}_{2,rough}$, methods such as grayscale centroid are used to estimate the precise value of ${\tilde{u}}_{2}$.

### 4.4. System Calibration

**Figure 7:**
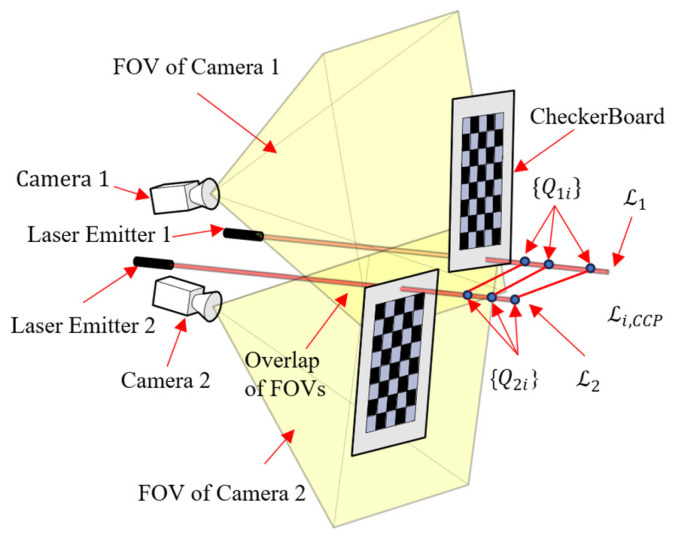
The two-step camera calibration method.

A two-step calibration method is adopted, as illustrated in Figure 7. Before applying this method, the intrinsic parameters of the cameras are calibrated using Zhang’s method. The two steps of the method are as follows:

**Step 1:** Collect the coordinate set of points on the laser, which includes the following sub-steps:

(1) A checkerboard and the Perspective-n-Point (PNP) algorithm are used to independently sample the spatial points on the laser within the FOV of each camera, obtaining a non-corresponding control point (NCCP) coordinate set ${\left\{NCCP_{ki}\right\}}^{\left(c_{n}\right)}$ under the $c_{1},c_{2}$ frames. Here, the subscript k represents the index of the laser, i represents the index of the coordinate in the set, and n=1,2. Using these NCCP sets, the camera–laser triangulation unit can be calibrated based on Equations (7) and (8).
(2) Using a flat plate, the corresponding control point (CCP) set ${\left\{CCP_{ki}\right\}}^{\left(c_{n}\right)}$ is obtained under $c_{1},c_{2}$ frames based on Equation (8).

**Step 2:** Estimate the camera pose, which includes the following sub-steps:

(1) According to Equation (5), a Plücker coordinate can be given by two three-dimensional points, thus N three-dimensional points give $N\left(N-1\right)/2$ Plücker coordinates. The NCCP coordinate set is used to obtain the NCCP–Plücker coordinate set ${\left\{L_{ki}\right\}}_{NCCP}^{\left(c_{n}\right)}$ for the k-th laser beam, where i is the index of the Plücker coordinate.
(2) The CCP coordinate set is used to obtain the Plücker coordinates of several spatial lines that are not parallel to the lasers. These are called CCP–Plücker coordinates ${\left\{L_{i}\right\}}_{CCP}^{\left(c_{n}\right)}$, where each coordinate in this set corresponds to a common line in the object space.
(3) The ${\left\{L_{ki}\right\}}_{NCCP}^{\left(c_{n}\right)}$ and ${\left\{L_{ki}\right\}}_{CCP}^{\left(c_{n}\right)}$ datasets are merged and input into the developed DQ-Laplacian maximum correntropy criterion (DLM) algorithm program to calculate the pose parameters of the two cameras.

This method requires both NCCP and CCP sets because the DLM algorithm requires the Plücker coordinates of at least two non-parallel lines, as explained in Section 4.5.2.

### 4.5. DLM Algorithm

The DLM algorithm uses the Laplace–MCC to model the fitting residuals and estimate the camera pose based on the Plücker coordinates of the corresponding lines in the two camera coordinate frames. In this section, the Laplace–MCC algorithm is introduced and the DLM algorithm is developed.

#### 4.5.1. Introduction of Laplace–MMC

To make this study self-contained, an overview of the Laplace–MCC algorithm is provided [53]. The cross entropy of two random variables and is given by the following:

(19) $$V_{\sigma }=E\left[\kappa_{\sigma }\left(Y-Z\right)\right]$$

where $\kappa_{\sigma }$ is the kernel function and σ is the size of the kernel function used to control the radius of influence.

For a discrete sample set, there is the following:

(20) $${\hat{V}}_{\sigma }\left(Y,Z\right)=\frac{1}{N}\sum_{i=1}^{N}\kappa_{\sigma }\left(Y_{i}-Z_{i}\right)$$

where $\kappa_{\sigma }\left(Y_{i}-Z_{i}\right)=\exp\left(-\left|Y_{i}-Z_{i}\right|/\sigma \right)$ is the Laplacian kernel.

**Figure 8:**
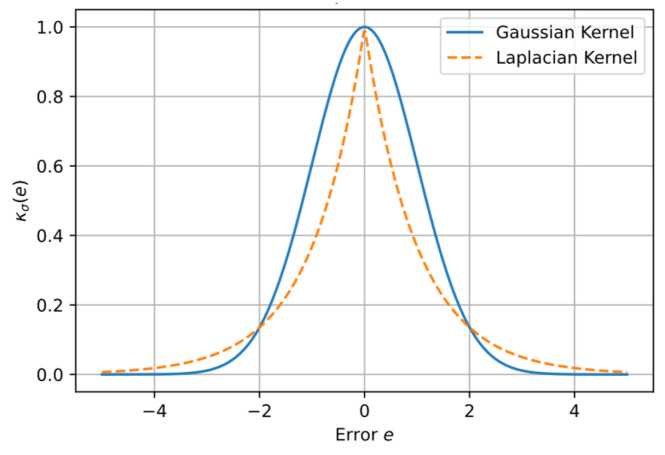
Gaussian vs. Laplacian kernels (σ=1).

As illustrated in Figure 8, the Laplacian kernel has a sharper central cusp and much heavier tail than the Gaussian kernel. This shape allows it to down-weight extreme outliers while still retaining useful information carried by moderate residuals. Consequently, the Laplacian kernel is well suited to heavy-tailed error distributions and datasets containing many moderate outliers—situations in which the Gaussian often over-penalizes residuals and degrades estimation accuracy. In our application, laser spots captured with a checkerboard typically exhibit this error pattern; numerous mid-level deviations persist after pre-processing, whereas gross outliers are largely removed, making the Laplacian kernel the more effective choice.

According to Equation (20), the optimization problem is given by the following:

(21) $$\underset{a}{\max}\frac{1}{N}\sum_{i=1}^{N}\exp\left(-\left|Y_{i}\left(a\right)-Z_{i}\right|/\sigma \right)$$

Note that $f\left(y\right)=e^{-y}$ has the following conjugate convex function:

(22) $$f\left(y\right)=\underset{\omega }{\sup}\left[\omega y-\phi (\omega )\right]$$

where the equation describes the maximum difference between the linear function ωy and $\phi \left(\omega \right)$, and this difference is maximized when $\omega =-f\left(y\right)$.

Substituting $y_{i}=\left|Y_{i}a-Z_{i}\right|/\sigma$ into Equation (22) causes the raw optimization problem to become the following:

(23) $$\underset{a,\omega }{\max}\sum_{i=1}^{N}\left(\omega_{i}\left|Y_{i}\left(a\right)-Z_{i}\right|/\sigma -\phi \left(\omega_{i}\right)\right)$$

This problem can be solved using an alternating iterative approach by breaking it down into the following two subproblems:

Subproblem 1:

(24a) $$\underset{a}{\max}\sum_{i=1}^{N}\left({\bar{\omega }}_{i}\left|Y_{i}\left(a\right)-Z_{i}\right|/\sigma \right)$$

Subproblem 2:

(24b) $$\underset{\omega }{\max}\sum_{i=1}^{N}\left(\omega_{i}\left|Y_{i}\left(\bar{a}\right)-Z_{i}\right|/\sigma -\phi \left(\omega_{i}\right)\right)$$

where Equation (24a) updates the model, and Equation (24b) updates the weight. The solution to Subproblem 2 is given by the following:

(25) $$\bar{\omega }=-\exp\left(-\left|Y_{i}\left(\bar{a}\right)-Z_{i}\right|/\sigma \right)$$

#### 4.5.2. DLM Derivation

The Plücker coordinates of the line L can be described using the following dual quaternions [47]:

(26) $$\begin{array}{ll} \hat{\dot{L}} & =\dot{L}+\epsilon {\dot{L}}_{O}\\ & =\left(L_{1}+\epsilon L_{O1}\right)i+\left(L_{2}+\epsilon L_{O2}\right)j+\left(L_{3}+\epsilon L_{O3}\right)k \end{array}$$

where ϵ is the dual operator, $\epsilon^{2}=0$, ϵ≠0, i, j, k are the basis elements of the quaternion, $\dot{L}$ is the real part of $\hat{\dot{L}}$, and ${\dot{L}}_{O}$ is the dual part. Both $\dot{L}$ and ${\dot{L}}_{O}$ are pure quaternions. The Euclidean transformation of the Plücker coordinates is given by the following:

(27) $$\hat{\dot{L^{\prime }}}={\hat{\dot{q}}}^{-1}\hat{\dot{L}}\hat{\dot{q}}={\dot{L}}^{\prime }+\epsilon {\dot{L}}_{O}^{\prime }$$

where $\left\|\hat{\dot{q}}\right\|=1$, and the following:

(28) $$\hat{\dot{q}}=\cos\left(\hat{\theta }/2\right)+\sin\left(\hat{\theta }/2\right)\hat{n}$$

(29) $${\hat{\dot{q}}}^{-1}=\cos\left(\hat{\theta }/2\right)-\sin\left(\hat{\theta }/2\right)\hat{n}$$

(30) $$\hat{\theta }=\theta +\epsilon S$$

(31) $$\hat{n}=\left[\left(L_{1}+\epsilon L_{O1}\right)i+\left(L_{2}+\epsilon L_{O2}\right)j+\left(L_{3}+\epsilon L_{O3}\right)k\right]$$

By separating the dual and non-dual parts, Equation (27) can be expanded as follows:

(32) $$\left\{\begin{array}{c} {\dot{L}}^{\prime }=\dot{q}\dot{L}{\dot{q}}^{-1}\;\;\;\;\;\;\;\;\;\;\;\;\;\;\;\;\;\;\;\;\;\;\;\;\;\;\;\;\;\;\;\;\;\;\;\;\;\;\\ {\dot{L}}_{O}^{\prime }=\dot{q}\dot{L}{\dot{q}}_{O}^{-1}+\dot{q}{\dot{L}}_{O}{\dot{q}}^{-1}+{\dot{q}}_{O}\dot{L}{\dot{q}}^{-1} \end{array}\right.$$

By right multiplying both sides by $\dot{q}$, the first sub-equation of Equation (32) becomes the following:

(33) $${\dot{L}}^{\prime }\dot{q}-\dot{q}\dot{L}=0$$

By right multiplying both sides by $\dot{q}$, and utilizing ${\dot{q}}^{-1}{\dot{q}}_{O}+{\dot{q}}_{O}^{-1}\dot{q}=0$, the second sub-equation becomes the following:

(34) $${\dot{L}}_{O}^{\prime }\dot{q}-\dot{q}{\dot{L}}_{O}+{\dot{L}}^{\prime }{\dot{q}}_{O}-{\dot{q}}_{O}\dot{L}=0$$

The computation of the quaternions can be represented by matrices. Equations (33) and (34) can be rewritten as follows:

(35a) $$\left(H_{{\dot{L}}^{\prime }}-M_{\dot{L}}\right)\dot{q}=0$$

(35b) $$\left(H_{{\dot{L}}_{O}^{\prime }}-M_{{\dot{L}}_{O}}\right)\dot{q}+\left(H_{{\dot{L}}^{\prime }}-M_{\dot{L}}\right){\dot{q}}_{O}=0$$

where

$$\begin{array}{cc} H_{\dot{q}} & =\left[\begin{array}{cc} q_{0} & -q^{T}\\ q & q_{0}I_{3}+{\left[q\right]}_{\times } \end{array}\right]\\ M_{\dot{q}} & =\left[\begin{array}{cc} q_{0} & -q^{T}\\ q & q_{0}I_{3}-{\left[q\right]}_{\times } \end{array}\right] \end{array}$$

Based on these formulas, the coordinate parameter transformation problem can be decomposed into the following two sub-optimization problems according to Equation (21):

(36a) $$\underset{\dot{q}}{\max}J_{1}\left(\dot{q}\right)=\sum_{i=1}^{N}\exp\left\{-{\left\|\Gamma_{i}\dot{q}\right\|}_{1}/\sigma \right\}\;\;\;\;\;\;\;\;$$

(36b) $$\underset{{\dot{q}}_{o}}{\max}J_{2}\left({\dot{q}}_{O}\right)=\sum_{i=1}^{N}\exp\left\{-{\left\|\Gamma_{i}{\dot{q}}_{O}+\gamma_{i}\right\|}_{1}/\sigma \right\}$$

(36c) $$s.t.\left\{\begin{array}{c} {\dot{q}}^{T}\dot{q}=1\\ {\dot{q}}^{T}{\dot{q}}_{O}=0 \end{array}\right.\;\;\;\;\;\;\;\;\;\;\;\;\;\;\;\;$$

where subscript i represents the index of Plücker coordinate, and the other parts are as follows:

(37) $$\Gamma_{i}=H_{{\dot{L}}_{i}^{\prime }}-M_{{\dot{L}}_{i}}$$

(38) $$\gamma_{i}=\left(H_{{\dot{L}}_{Oi}^{\prime }}-M_{{\dot{L}}_{Oi}}\right)\dot{q}$$

Equations (36a,b) can be transformed into an epigraph form according to Equation (24a), with the constraints in Equation (36c) resulting in the following two linear programming subproblems:

Subproblem 1:

(39) $$\begin{array}{c} \underset{x}{\min}J_{1}^{\prime }\left(x,{\bar{\omega }}_{1}\right)=c^{T}\left(\bar{\omega }\right)x\\ s.t.\left\{\begin{array}{c} \left|\tilde{\Gamma }x\right|\leq \Lambda x\\ a^{T}x=1 \end{array}\right.\;\;\;\;\;\;\;\;\;\;\;\;\;\;\;\;\;\; \end{array}$$

Subproblem 2:

(40) $$\begin{array}{c} \underset{y}{\min}J_{2}^{\prime }\left(y,\bar{\dot{q}},{\bar{\omega }}_{2}\right)=c^{T}\left(\bar{\omega }\right)y\\ s.t.\left\{\begin{array}{c} \left|\tilde{\Gamma }y+\gamma \right|\leq \Lambda y\\ b^{T}\left(\bar{\dot{q}}\right)y=0 \end{array}\right.\;\;\;\;\;\;\;\;\;\;\;\;\;\;\; \end{array}$$

where ${\bar{\omega }}_{1}$ and ${\bar{\omega }}_{2}$ are weight vectors of length 4N, τ and η are slack vectors of length 4N, $\mathrm{vstack}\left(\cdot \right)$ represents the vertical stacking operator for the matrices, and the other symbols are as follows:
$\;\;x={\left[\begin{array}{cc} {\dot{q}}^{T} & \tau^{T} \end{array}\right]}^{T}$$y={\left[\begin{array}{cc} {\dot{q}}_{O}^{T} & \eta^{T} \end{array}\right]}^{T}$$\;\;\;\tilde{\Gamma }=\left[\begin{array}{cc} \Gamma & 0_{4N\times 4N} \end{array}\right]$$\;\;\;\Gamma =vstack\left(\Gamma_{1},\ldots ,\Gamma_{N}\right)$$\;\;\;\Lambda =\left[\begin{array}{cc} 0_{4N\times 4} & I_{4N} \end{array}\right]$$a=\left[\begin{array}{cc} 1 & 0_{3+4N}^{T} \end{array}\right]$$b^{T}\left(\bar{\dot{q}}\right)={\left[\begin{array}{cc} \bar{\dot{q}} & 0_{4N} \end{array}\right]}^{T}$$\;\;\gamma ={\left[\begin{array}{ccc} \gamma_{1}^{T} & \ldots & \gamma_{N}^{T} \end{array}\right]}^{T}$$\;\;c\left(\bar{\omega }\right)={\left[{\begin{array}{cc} 0_{4}^{T} & \bar{\omega } \end{array}}^{T}\right]}^{T}$


The function of the second constraint $a^{T}x=1$ in Equation (39) is equivalent to ${\dot{q}}^{T}\dot{q}=1$ in Equation (36c), thus avoiding the need to solve a non-convex optimization problem. This equivalence follows from Theorem 1 (see Appendix A for proof). According to the proposition, and noticing that ${\dot{q}}^{T}\dot{q}=1$, a unique solution for $\Gamma \dot{q}=0$ exists. Given that Equation (39) essentially solves $\Gamma \dot{q}=0$, Equations (39) and (40) have a unique solution only when the condition in Theorem 1 is satisfied.

**Theorem** **1.** *For matrix Γ, if the number of lines involved in the calculation is greater than 2 and the directions of these lines are not completely the same, then the rank of Γ is 3, indicating that the corresponding null-space dimension of Γ is 1. Otherwise, the rank of Γ is 2, and the null space dimension is 2.*

After calculating x and y according to Equations (39) and (40), the estimated values of $\dot{q}$ and ${\dot{q}}_{O}$ is given by the following:

(41a) $$\hat{\dot{q}}=\eta x/{\left\|\eta x\right\|}_{2}$$

(41b) $${\hat{\dot{q}}}_{O}=\eta y$$

where $\eta ={\left[\begin{array}{cc} 1_{1\times 4} & 0_{1\times 4N} \end{array}\right]}^{T}$.

Based on the derivation above, the DLM algorithm is presented in Algorithm 1. To improve the stability and accuracy of the results, the algorithm uses a *modified Silverman’s rule* (MSR) to update the Laplace kernel size σ, given by the following:

(42) $$\sigma =\left\{\begin{array}{c} 0.8\times E_{max}\times E_{IQR}\times D_{S}^{-1},\;\;\;\;\;\;\;\;\;\;\;\;\;\;\;\;\;\;\;\;\;\;\;\mathrm{if}\;D_{s}\leq 1.5\times E_{IQR}\\ 1.06\times \min\left\{\sigma_{E},p_{25}/1.34\right\}\times N^{-0.2},\;\;\;\;\;\;\;\;\;\;\;\;\;\;\mathrm{other}\;\;\;\;\;\;\;\;\;\;\;\;\;\;\; \end{array}\right.$$

where $E_{\max}$ is the maximum fitting residual, $E_{\mathrm{IQR}}$ is the interquartile range of the fitting residuals, $D_{S}=\left|\sigma_{E}-E_{\mathrm{median}}\right|$ represents the asymmetry of the fitting residual distribution, and $E_{\mathrm{median}}$ is the median of the fitting residuals. The MSR prevents the value of σ from being too small, which would distort the results.

$\dot{q}$ and ${\dot{q}}_{O}$ can be converted into a rotation matrix and translation vector, respectively, which can be substituted into Equations (15) and (16) for laser spot searching. The conversion formulas are as follows [54]:

(43a) $$R=\left[\begin{array}{ccc} 2\left(q_{0}^{2}+q_{1}^{2}\right)-1 & 2\left(q_{1}q_{2}-q_{0}q_{3}\right) & 2\left(q_{1}q_{3}+q_{0}q_{2}\right)\\ 2\left(q_{1}q_{2}+q_{0}q_{3}\right) & 2\left(q_{0}^{2}+q_{2}^{2}\right)-1 & 2\left(q_{2}q_{3}-q_{0}q_{1}\right)\\ 2\left(q_{1}q_{3}-q_{0}q_{2}\right) & 2\left(q_{2}q_{3}+q_{0}q_{1}\right) & 2\left(q_{0}^{2}+q_{3}^{2}\right)-1 \end{array}\right]$$

(43b) $$t=2{\dot{q}}_{O}{\dot{q}}^{-1}=2M^{T}\left(\dot{q}\right){\dot{q}}_{O}$$

**Algorithm 1. DLM-MSR Algorithm****Input:**${\left\{L_{i}\right\}}^{\left(c_{1}\right)}$: A set of Plücker coordinates of at least 2 non-parallel lines in the $c_{1}$-Frame;${\left\{L_{i}^{’}\right\}}^{\left(c_{2}\right)}$: The corresponding set of Plücker coordinates in the $c_{2}$-Frame;K: The maximum number of iterations for the solver;E: The minimum update step size;**Process:**0: Initialize $\omega^{T}\left(1\right)=\left[-1,-1,\ldots ,-1\right]$, $z^{T}\left(0\right)=\left[0_{8}\right]$;**for** k=1:K1:Set $\bar{\omega }=\hat{\omega }\left(k\right)$, compute x(k) according to Equation (39) and $\dot{q}\left(k\right)$ according to Equation (41a);
2:Set $\bar{\dot{q}}=\hat{\dot{q}}\left(k\right)$ and substitute it into Equation (40) to compute y(k), then computer ${\hat{\dot{q}}}_{O}\left(k\right)$ according to Equation (41b)3:Set $z^{T}(k)=\left[\begin{array}{cc} \hat{\dot{q}}\left(k\right) & {\hat{\dot{q}}}_{O}\left(k\right) \end{array}\right]$ to update the result;4:If $\left\|z(k)-z(k-1)\right\|<E$ or k=K, then break;5:Update the kernel size σ using Equation (42), and compute $\omega \left(k+1\right)$ using Equation (25);**end for****Return:**$\hat{\dot{q}}$ and ${\hat{\dot{q}}}_{O}$


## 5. Experiment and Discussion

The content of this section is as follows:

(1) Section 5.1, Section 5.2 and Section 5.3 present a numerical simulation, an indoor test, and an outdoor field test that collectively evaluate the performance of the DLM algorithm. The simulation was implemented in Python 3.10, and the optimization problems in Equations (39) and (40) were solved with the CVXPY library (ver. 1.5.2).
(2) In Section 5.4, the DLM algorithm was used to calibrate the RIC, and actual RTL images were collected to verify the feasibility of the proposed laser-assisted image stitching method. The experimental data were processed with custom Python scripts, OpenCV 4.9.0 was used for fundamental image operations, and the Pywt library (ver. 1.7.0) was employed for wavelet analysis.

### 5.1. DLM Simulation

**Figure 9:**
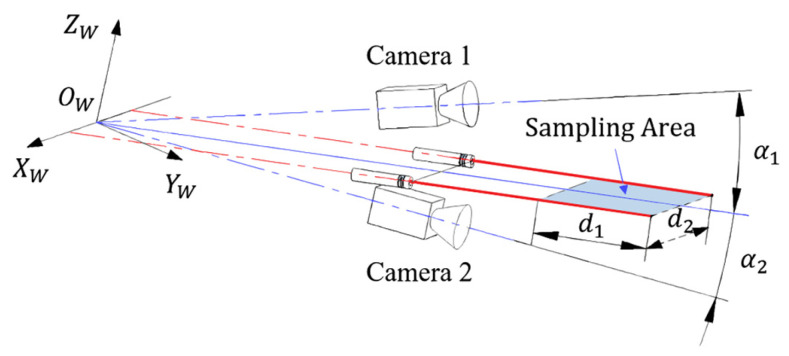
DLM simulation diagram.

As shown in Figure 9, the simulation model consists of two cameras and two lasers. The lines representing the laser beams are labeled as $L_{1}$ and $L_{2}$, and their directions are parallel. The optical axes of the lasers and cameras are both perpendicular to the $X_{W}$-axis, and the angles between the optical axes of Cameras 1 and 2 and the laser beams are $\alpha_{1}$ and $\alpha_{2}$, respectively. In the rectangular “Sampling Area” shown in the figure, the points on the lasers are evenly sampled at intervals of $\Delta d_{1}$. The sampling noise is assumed to follow a Gaussian distribution, with the standard deviation of the sampling noise being $\sigma_{1}$ at the nearest point to the camera and $\sigma_{2}$ at the farthest point. The standard deviation of the sampling noise between the nearest and farthest points is given by $\sigma (d)=d\left|\sigma_{2}-\sigma_{1}\right|/d_{1}$, where d is the distance from the sampling location to the nearest point.

In the simulation set $d_{1}=1000$ mm, $d_{2}=300$ mm, $\Delta d_{1}=50$ mm, $\alpha_{1}=\alpha_{2}=8{}^{\circ}$, $\sigma_{1}=1$ mm, and $\sigma_{2}=2$ mm. A total of 42 points were sampled from $L_{1}$ and $L_{2}$ in each camera’s coordinate frame. Two NCCP-CCP mixed Plücker coordinate sets ${S^{\left(c_{k}\right)}=\left\{L_{ij}\;|\;i\neq j=1,\ldots ,21\right\}}^{\left(c_{k}\right)}$, k=1,2, were obtained, and $\left|S^{\left(c_{1}\right)}\right|\equiv \left|S^{\left(c_{2}\right)}\right|=861$, where $\left|S\right|$ denotes the number of members in the set S.

To simulate the deviation caused by ambient light, some of the samples were randomly selected and larger Gaussian noise was added to make them outliers, with a standard deviation of $\sigma_{3}=4.58$ mm. The experiment was divided into six groups, simulating calibration results when outliers accounted for 0%, 5%, 10%, 15%, 20%, and 25% of the sample set. Each experiment was repeated 300 times. During the experiment, the maximum number of iterations for the DLM algorithm was set to K=100, and the minimum update step was $E=10^{-10}$.

The DQ-LS algorithm [49] was used as a comparison method, and the following three variants of the DLM algorithm were tested: DLM-MSR (DLM with Modified Silverman’s rule), DLM-SR (DLM with raw Silverman’s rule), and DM (DLM without the weight update step). Among these, DLM-MSR is the algorithm described in Algorithm 1, DLM-SR uses the original Silverman’s rule to update the kernel size, and the DM algorithm eliminates the weight update step.

The experimental results are listed in Table 3. The table shows the average L2 norm of the estimation error of Euler angle (EEA) and the estimation error of translate (ET). In addition, it shows the average number of iterations required for DLM-MSR and DLM-SR to satisfy the convergence conditions.

The results indicated that the DQ-LS method is extremely sensitive to noise and outliers. As the proportion of outliers increased from 0% to 25%, the EEA of the DQ-LS algorithm increased by 0.0062 (about 98%) and ET increased by about 21.85 (about 177%). The performance of the DLM-SR algorithm is inferior to that of the DM algorithm, suggesting that the raw Silverman’s rule prevents the algorithm from converging. In contrast, the DLM-MSR algorithm had the best estimation accuracy. As the proportion of outliers increased from 0% to 25%, the EEA increased by only 0.0011 (about 44%) and the ET increased by only 1.63 (about 45%). Furthermore, the average number of iterations required for convergence was about 4, significantly fewer than the iterations required by the DLM-SR, demonstrating that the modified Silverman’s rule can significantly enhance the accuracy and efficiency of the algorithm.

### 5.2. Indoor Experiment

**Figure 10:**
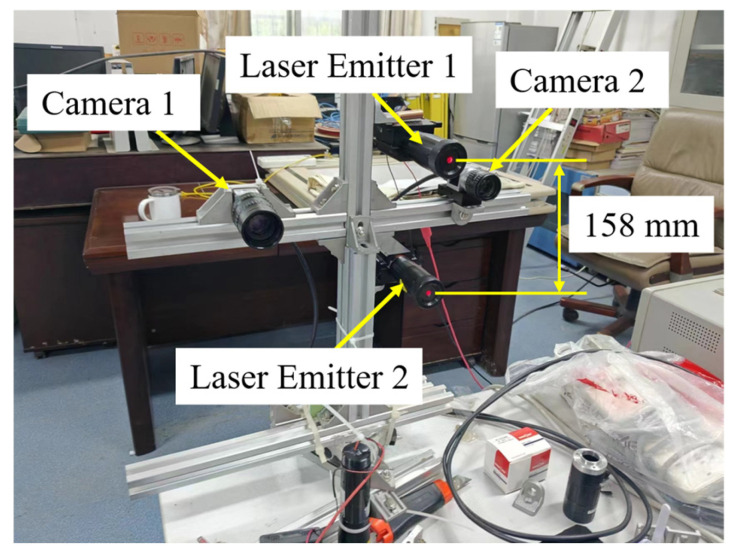
Picture of experimental platform.

An indoor experiment was carried out to validate the proposed method. The experiment platform was equipped with two Balser acA2440-75um cameras, each with an f = 25 mm lens, as shown in Figure 10. The lasers were adjusted to be nearly parallel to simulate the worst condition. During the experiment the common FOV of the cameras was only minimally restricted, allowing the use of the EPNP algorithm [55] to estimate the camera pose parameters, which were then compared with the results estimated by other algorithms. The experiment followed the calibration method described in Section 4.4, and the camera pose parameters were estimated using DQ-LS, DLM-MSR, DLM-SR, DM, and EPNP.

During the experiment, the NCCP sets collected from lasers 1 and 2 under the $c_{1}$-Frame and $c_{2}$-Frame were denoted as $NCCP_{ij}$, and the CCP set as $CCP_{i}$, where i,j=1,2, with i representing the camera coordinate system index and j representing the collimation laser index. The numbers of collected NCCPs was as follows:

$$\left|NCCP_{11}\right|=480\;\;\left|NCCP_{12}\right|=549$$

$$\left|NCCP_{21}\right|=887\;\;\left|NCCP_{22}\right|=954$$

The corresponding number of NCCP–Plücker coordinates was 229920, 300852, 785882, and 909162, respectively. The number of collected CCP pairs was four, corresponding to four CCP–Plücker coordinates. The number of CCPs involved in this experiment was relatively small, because for the real equipment collecting CCPs requires a large flat plate which must be suspended in the air, making it difficult to move. Excessive CCPs would pose additional challenges for the production of the equipment.

To ensure balanced sample numbers, the following preprocessing was applied:

(1) A minimum point pair distance of 500 mm was used to eliminate Plücker coordinates generated by closely spaced point pairs in the NCCP–Plücker set;
(2) A total of 500 Plücker coordinates were randomly selected from the filtered NCCP–Plücker set for calculation;
(3) The chosen NCCP–Plücker coordinates with the CCP–Plücker coordinates were used to obtain the set used for pose estimation.

**Figure 11:**
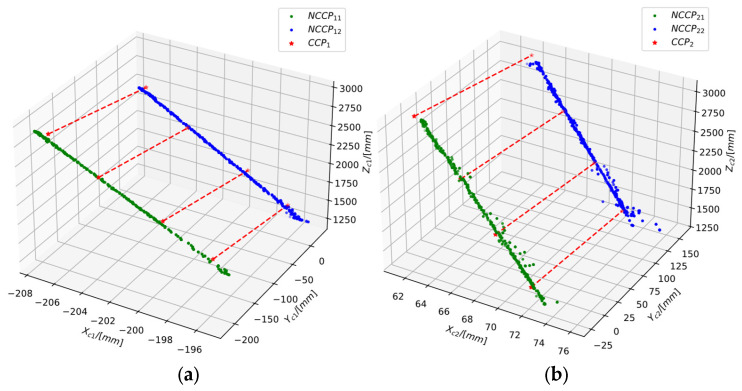
Distribution of NCCPs and CCPs involved in calculation. (**a**) Control points in c1-Frame; (**b**) control points in c2-Frame (The red dashed lines in the figure represent the connections between the NCCPs).

The distributions of NCCPs and CCPs in the two camera coordinate frames after the above processing are shown in Figure 11.

Table 4 lists the estimated camera pose parameters from different methods, as well as the mean error from the reprojection of the CCP set. It also provides the *root mean square* (RMS) of the epipolar constraint deviation $e_{ep}=l_{ep}^{T}\tilde{u}$, calculated using the CCP coordinate set according to Equation (16). The RMS is given by the following:

(44) $$\mathrm{RMS}\left(X\right)=\sqrt{N^{-1}\sum_{i=1}^{N}x_{i}}$$

According to the reprojection error of the CCPs and the epipolar lines, it is evident that the performances of DQ-LS, DLM-MSR, and DM are similar and better than those of EPNP. Furthermore, the results obtained using the DLM-SR method are severely distorted, indicating that the original Silverman’s rule fails to provide a reasonable Laplace kernel size.

**Figure 12:**
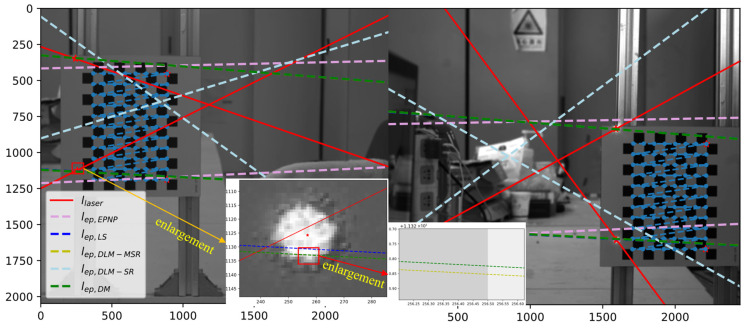
Epipolar projection on image.

Figure 12 shows the projection of the constrained epipolar lines from the different algorithms. It can be observed that $l_{ep,\mathrm{LS}}$, $l_{ep,\mathrm{DLM}-\mathrm{MSR}}$, and $l_{ep,\mathrm{DM}}$ are very similar, and their RMS values are also very similar, whereas $l_{ep,\mathrm{DLM}-\mathrm{SR}}$ significantly deviates from the laser spot.

**Figure 13:**
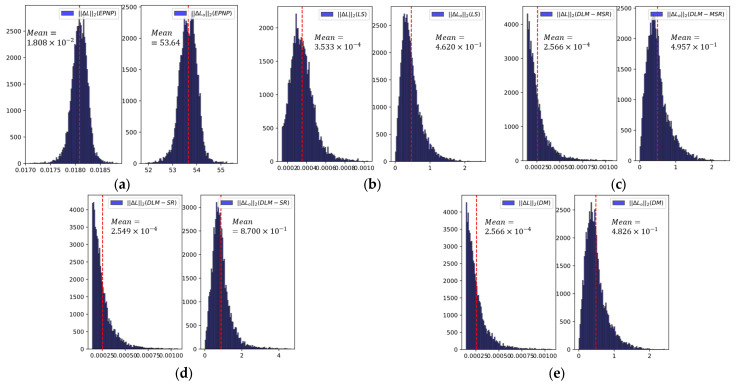
Histogram of reprojection errors for NCCP–Plücker coordinates. (**a**) EPNP; (**b**) DQ-LS; (**c**) DLM-MSR; (**d**) DLM-SR; (**e**) DM. In every sub-panel, the left histogram shows the reprojection error of the line direction vectors, while the right histogram shows the reprojection error of the line moment vectors.

The camera pose estimation results of each algorithm were further analyzed using the reprojection error of the NCCP–Plücker coordinate set. Because the complete $NCCP_{ij}$–Plücker coordinate set is large, the mean of this set is statistically close to the true value, which can be used as a reference benchmark. Figure 13 shows a histogram of the L2 norm of the reprojection error. It can be seen that the reprojection errors of the DQ-LS, DLM-MSR, and DM methods were small and similar.

These comparison results indicate that DQ-LS, DLM-MSR, and DM have similar estimation accuracies in this case, but in terms of pose angle estimation DLM-MSR and DM can provide more accurate results. The performances of the DLM-MSR and DM algorithms were similar, likely because of the low number of outliers in the experimental sample.

### 5.3. Outdoor Experiment

**Figure 14:**
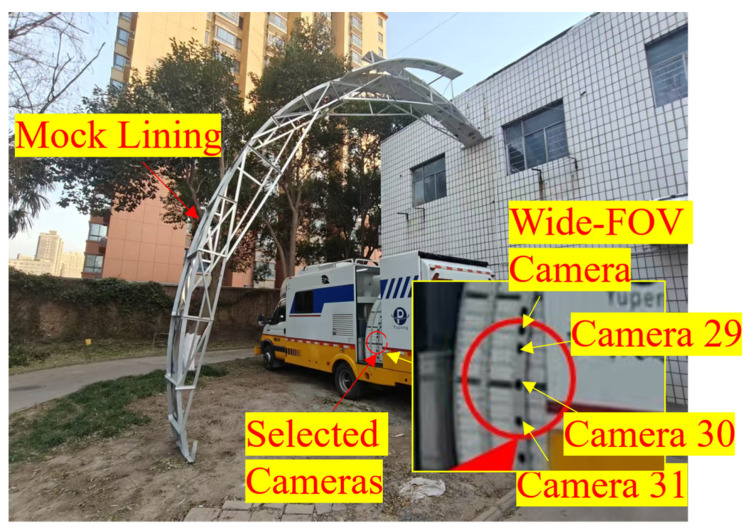
Outdoor experiment site.

**Figure 15:**
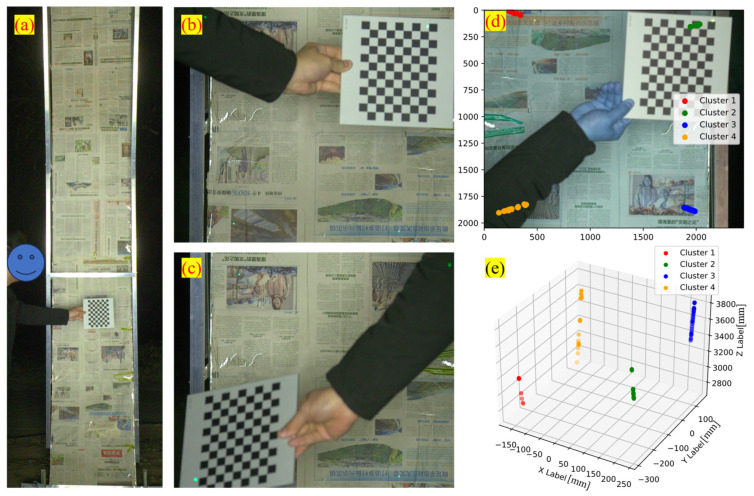
Typical experiment images. (**a**) Frame captured by the wide-FOV camera; (**b**,**c**) frames captured by two narrow-FOV cameras; (**d**) projection of the laser spot trajectories in image space; (**e**) three-dimensional positions of the laser spots in the corresponding camera frame. In panels (**c**,**d**) the spot sets are numbered clockwise, starting from the upper-left laser, and are color-coded red → green → blue → orange.

The proposed method was applied to calibrate the side wall RTL subsystem. The camera highlighted in Figure 14 was selected, and several representative images are shown in Figure 15. During calibration the lens was focused on the mock tunnel lining to help the operator locate the laser spots. As a consequence, the checkerboard appears slightly out of focus in Figure 15c, which inevitably reduces the accuracy of spot localization.

During the experiment each camera operated at 33 fps while the operator moved the calibration board to intercept the laser spots. Extrinsic parameters were estimated for the three adjacent side wall cameras (IDs 29, 30, 31). The following four calibration strategies were evaluated: DLM-MSR, DM, DQ-LS, and EPNP.

For DLM-MSR, DM, and LS the numbers of NCCPs entering the computation were 140, 131, and 157, respectively, plus a single pair of CCPs. For the EPNP route 703, 514, and 855 checkboard image pairs were available; a pose was first computed for each pair and the mean pose was taken as the final estimate. As indicated in Figure 15c,d, point sets 1/4 constitute one set of conjugate features for adjacent cameras, whereas point sets 2/3 form the second set.

**Figure 16:**
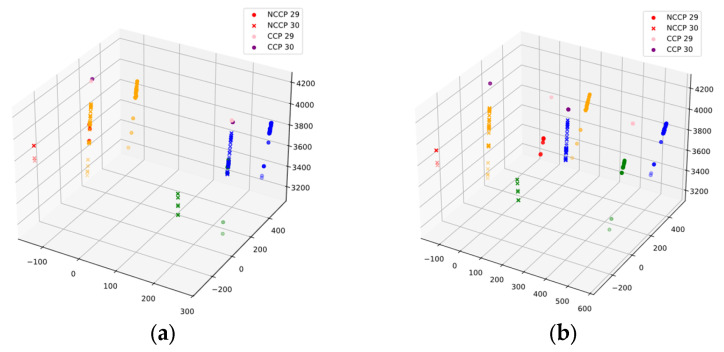
Re-projection of control points in three-dimensional space. (**a**) Result obtained with the DLM-MSR/DLM algorithms; (**b**) result obtained with the LS algorithm. The color of each laser spot cluster is identical to that used in Figure 15.

The results are summarized in Table 5 and the nominal installation angles are listed for reference. Convergence is assessed by comparing to the design angles. EPNP diverges markedly, showing that the wide-FOV-based strategy is unsuitable—most likely because the lower resolution of the wide-FOV images inhibits accurate corner localization. In contrast, DLM-MSR, DM, and LS all return angles close to the design values, with DLM-MSR and DM virtually identical. The three-dimensional reprojection plots in Figure 16 corroborate these findings. With DLM-MSR or DM the re-projected NCCP and CCP laser spots fall on a common straight line, whereas the LS solution fails to align the four laser tracks, indicating that LS breaks down under the present imaging conditions.

The numerical differences between DLM-MSR and DM are negligible. This is most likely because the mean shift pre-filter applied during calibration had already removed the majority of outliers, leaving both solvers with virtually the same inlier set. To magnify any residual discrepancy, the laser spot coordinates obtained from each solution were re-projected into three-dimensional space, a best-fit Plücker line was computed, and the perpendicular distance of every point to that line was evaluated as follows:

(45) $$d={\left[r^{T}r-{\left(r^{T}L\right)}^{2}\right]}^{0.5}$$

where L and $L_{O}$ are the Plücker coordinates of the fitted line, $\left\|L\right\|=1,r=P-{\left[L\right]}_{\times }L_{O}$, and P is the coordinate of a three-dimensional point.

For each camera pair the RMS and mean absolute error (MAE, Equation (46)) of the distances are listed in Table 6. The 29/30 pair exhibits larger residuals than the 30/31 pair because its NCCP set is smaller and therefore has a higher noise fraction. Within each pair, RMS and MAE are almost identical, indicating an outlier-free dataset.

For cameras 29/30, DLM-MSR reduces the projection error by ≈0.9 mm with respect to DM, whereas for cameras 30/31 the two algorithms are statistically indistinguishable. Hence the MSR weight-updating mechanism does not provide a meaningful accuracy gain in this scenario; the non-iterative DM variant achieves comparable accuracy with lower computational cost. In practice, whether to activate the MSR re-weighting step should be decided adaptively according to the noise characteristics of the dataset.

(46) $$MAE\left(X\right)=N^{-1}\sum_{i=1}^{N}\left|x_{i}\right|$$

### 5.4. Image Stitching Experiment Based on Real RIC Data

#### 5.4.1. Selected Cameras

Cameras 5 and 6 of the top lining measurement system mounted on the RIC were selected for the experiment.

First, the intrinsic parameters of the cameras were calibrated using Zhang’s method. Then, the cameras and laser emitters were mounted on the vehicle. Finally, the calibration boards were suspended on a long pole to calibrate the camera–laser triangulation measurement units and camera pose parameters. The calibration results are listed in Table 7, where the Plücker coordinates of each laser were estimated using the least squares method based on the NCCP datasets. The intrinsic parameters of cameras were obtained using Zhang’s method, and the camera pose parameters were calculated using the DLM-MSR algorithm.

In the experiment, four pairs of CCPs were collected, and the numbers of NCCP samples were as follows:

$$\left|NCCP_{11}\right|=417\;\;\left|NCCP_{12}\right|=328$$

$$\left|NCCP_{13}\right|=371\;\;\left|NCCP_{14}\right|=484$$

$$\left|NCCP_{21}\right|=234\;\;\left|NCCP_{22}\right|=445$$

$$\left|NCCP_{23}\right|=543\;\;\left|NCCP_{24}\right|=453$$

**Figure 17:**
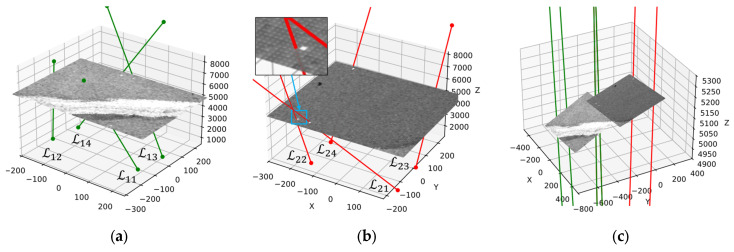
Object–space projection of laser and camera image. (**a**) Camera 1 image and the projection of the laser in the $c_{1}$-Frame; (**b**) Camera 2 image and the projection of the laser in the $c_{2}$-Frame; (**c**) the projection of both camera images and lasers in the $c_{2}$-Frame (In the figure, the green lines denote the laser beams expressed in the $c_{1}$-frame, whereas the red lines denote the beams expressed in the $c_{2}$-frame).

The subscripts of the NCCP datasets correspond to the laser indices shown in Figure 17.

The calibration results are shown in Figure 17. From Figure 17b, it can be seen that owing to vibration or calibration errors, some laser spots may deviate slightly from the expected positions; therefore, Equations (12) and (13) are needed for precisely images alignment.

#### 5.4.2. RTL Image Mosaicking with Laser Aid

**Figure 18:**
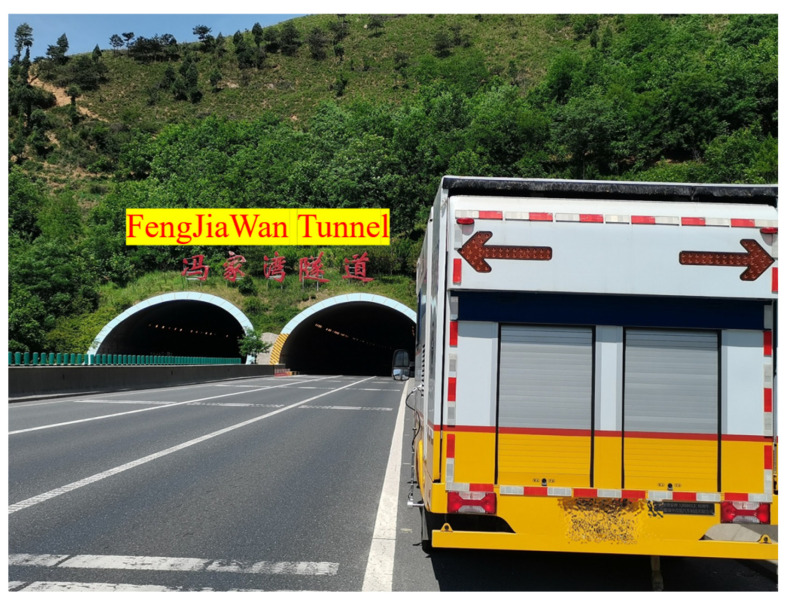
Experimental site.

**Figure 19:**
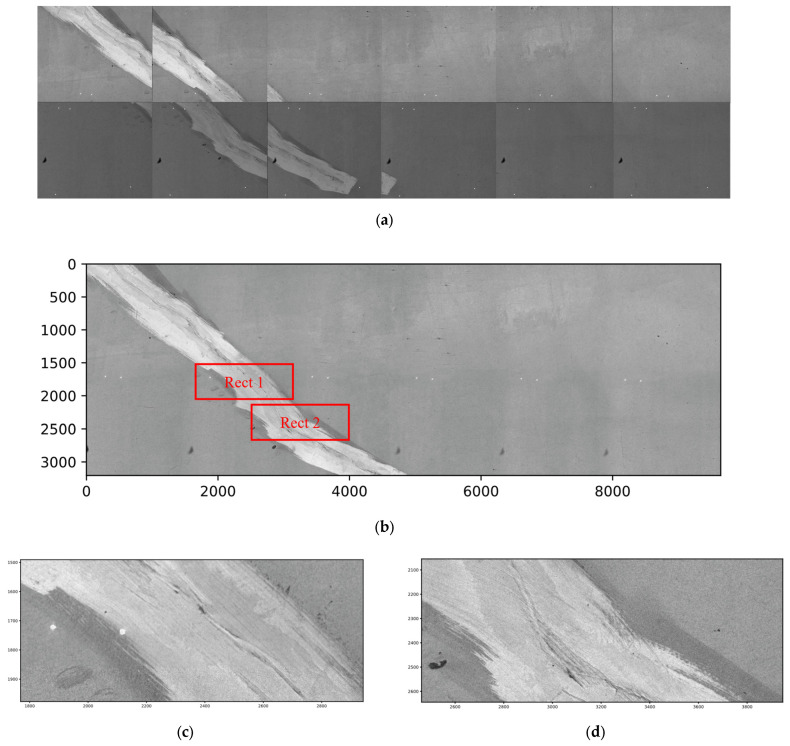
Tunnel lining image stitching results. (**a**) Raw images; (**b**) stitched image; (**c**) magnified view of Rect 1; (**d**) magnified view of Rect 2.

A tunnel located on the G30 National Highway in Xi’an, Shaanxi Province, China, was selected for side testing. The site images are shown in Figure 18. The collected images were stitched using the method described in Section 4.2, and stitching results are shown in Figure 19. According to Figure 19b, there are no significant stitching seams in the stitched images at the macro level. However, some faint shadow areas can be observed near the stitching seams, caused by the attenuation of camera illumination, which leads to a decrease in brightness on both sides of the image and can be corrected through flat-field correction.

From Figure 19c,d, in the locally magnified images it is difficult to observe obvious stitching seams, and the image texture near the seams maintains good continuity even though there are few corresponding features on the tunnel lining image. This shows that the laser spot-assisted registration method is efficient for obtaining high-quality panoramic images of tunnel linings.

#### 5.4.3. Comparison of RTL Image-Stitching Methods

To evaluate whether the laser-assisted stitching method proposed in this study genuinely improves registration accuracy, we benchmarked it against two widely used baselines—Windows Image Composite Editor (ICE 2.0.3) and the SIFT + RANSAC [56].

**Figure 20:**
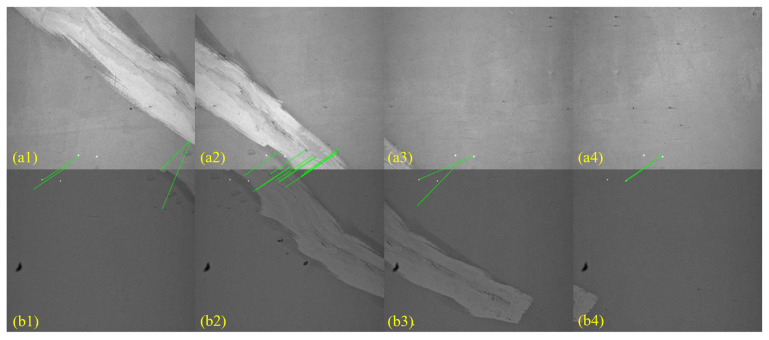
SIFT + RANSAC image registration results. (**a1**–**a4**) Images captured by Camera 5; (**b1**–**b4**) images captured by Camera 6.

**Figure 21:**
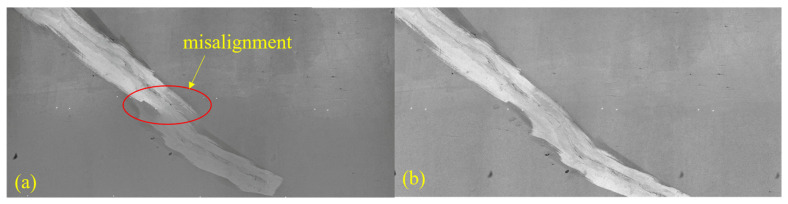
Comparison between ICE and the proposed method. (**a**) Mosaic produced by ICE; (**b**) mosaic produced by the proposed laser-assisted approach.

After undistortion and scale normalization with the calibration parameters, the RTL image pairs shown in Figure 20 were processed. The SIFT + RANSAC method produced reliable transformations only when a conspicuous crack spanned the overlap region (Figure 20(a2,b2)); for the remaining low-texture images, removing laser spot correspondences left either too few inliers to support a model or produced large geometric errors. Figure 21 contrasts the RTL mosaics generated by ICE and by our method. The ICE result exhibits conspicuous misalignment near the lining cracks, and the central laser spot trace deviates markedly from a straight line, indicating an unstable registration, whereas the mosaic produced by the proposed method preserves an almost perfectly straight laser line that coincides with the vehicle trajectory. These findings demonstrate that the laser-assisted method delivers a significant improvement in stitching accuracy for RTL imagery, a scenario in which the scarcity of natural texture limits conventional feature-based approaches.

## 6. Conclusions

This work addresses the dual goals of enhancing the quality and efficiency of RTL image acquisition. We introduce a collimated laser-assisted mobile scanning framework in which laser arrays supply artificial tie lines that (i) stabilize image stitching and (ii) provide an explicit mapping between individual camera images and the tunnel lining surface. To calibrate the camera array, we propose a single-checkerboard procedure that treats the laser lines as line correspondences, thereby determining the pose of each camera. To guarantee robustness, we develop a dual-quaternion Laplacian kernel-based algorithm (DLM-MSR) and establish the theoretical conditions under which this algorithm converges. Extensive simulations—as well as controlled indoor and field experiments—demonstrate that DLM-MSR achieves rapid convergence, delivers higher calibration accuracy than conventional methods, and remains stable even in the presence of substantial outlier noise.

A tunnel inspection prototype vehicle has been built on the basis of the proposed methodology. The paper presents a detailed hardware framework of this platform, including its principal sensors and computers together with their key performance specifications. These disclosures are intended to serve as a practical reference for the design and construction of similar inspection systems.

The performance of the proposed DLM-MSR algorithm was validated through both numerical simulation and physical experiments. Empirically, the solver converged in only 3~4 iterations on average and exhibited noticeably greater robustness. In Monte Carlo simulation, increasing the outlier ratio from 0% to 25% caused the DLM-MSR solution to degrade by only ~44% in Euler-angle error and ~45% in translation error, whereas competing algorithms deteriorated far more severely. Consistent results were obtained in laboratory and field trials: DLM-MSR delivered significantly higher calibration accuracy for the tunnel lining scanning system and completely avoided the divergence occasionally observed with the benchmark techniques.

An experiment using real RIC data confirmed the feasibility of the proposed RTL scanning method, and a comparative evaluation against conventional image-stitching techniques showed that the RTL mosaics produced by our approach achieve markedly higher registration accuracy than those obtained with traditional methods.

There are also the following drawbacks in this paper:

(1) This study assumes that the projection relationship between the camera images and tunnel lining is a planar projection, ignoring the curvature of the tunnel cross-section. Therefore, the laser array-assisted image stitching method proposed in this study is only applicable to tunnels with smooth cross-sectional profiles and may fail for tunnels with non-smooth cross-sectional profiles, such as immersed tube tunnels.
(2) This study did not address the issue of image stitching when laser points are missing.
(3) Because of the limitations of the current experimental conditions, this study did not analyze the pixel scale error in the stitched lining images, and the panoramic RTL image was not provided in this study.
(4) Because insufficiently diverse set of real-world RTL images, quantitative stitching-error statistics are not yet available.

We will investigate these issues in future research.

## Figures and Tables

**Table 1:** Representative RICs.

RIC System	Camera Type	Illumination	Auxiliary Sensor	Manufacturer Location
ZOYON TFS [14,15,16,17,18]	ASC	LED	Lidar	China
tjgeo TDV-H [19]	ASC	LED	Lidar	China
TiDS [20]	LSC	LSL	Lidar	China
Keisokukensa Co., MIMM-R [21]	ASC	LED	Lidar	Japan
Tonox TC-2 [22]	LSC	LSL	\	Japan
Ricoh TMS [23]	LSC	LSL	\	Japan
NEXCO Smart-EAGLE [24,25]	LSC	LED	\	Japan
Tunnel Tracer [26]	ASC	LED	\	Japan
Kim’s [27]	ASC	LED	\	South Korea
Nguyen’s [28,29]	ASC	LED	\	Japan
Alpha-product FOCUSα-T [30]	ASC	LED	Collimated lasers	Japan
Zou’s [31]	LSC	LED	Lidar	China
Tongji University’s [32]	ASC	LED	Lidar	China

Remark: ASC represents area-scanning camera, LSC represents line-scanning camera, LSL represents line-scanning laser, LED represents light-emitting diode. The symbol “\” indicates that the device is not equipped with the corresponding component.

**Table 2:** Main component specifications.

Component Type	Model	Key Parameters	Quantity	
Collimated laser	520 nm collimated laser	Output power 70 mW	Top: 22	Side: 44
LED strobe module	In-house design	18 × 18 W LED chips per module	Top: 120	Side: 160
Frequency divider	In-house design	FPGA: Altera EPF10K20TC144-4	single	
Server computer	Advantech AIIS-3410U	Intel i7-6700 CPU, 8 GB RAM	Top: 3	Side: 8
Narrow-FOV camera	Basler acA2440-75 um/uc	2440 × 2048 px, 3.45 µm pixel; lens focal length: *f* = 50 mm (side), *f* = 75 mm (top)	Top: 11 (Mono)	Side: 21 (Color)
Wide-FOV camera	Basler acA2440-75 uc	Same sensor as above; lens focal length: *f* = 8 mm	Top: 1	Side: 3

**Table 3:** Simulation result.

		Group 1	Group 2	Group 3	Group 4	Group 5	Group 6
DQ-LS	EEA	0.0063	0.0063	0.0080	0.0093	0.0112	0.0125
	ET	12.28	16.61	21.32	25.22	30.60	34.13
	CT	0.67	0.76	0.69	0.70	0.69	0.67
DLM-MSR	EEA	0.0025	0.0026	0.0030	0.0031	0.0035	0.0036
	ET	3.55	3.71	4.11	4.38	4.83	5.18
	IC	3.39	3.57	3.76	3.88	3.99	3.84
	CT	4.88	5.42	5.42	5.52	4.93	4.34
DLM-SR	EEA	0.0029	0.0030	0.0031	0.0034	0.0038	0.0040
	ET	3.99	4.2203	4.5065	4.7390	5.2032	5.6193
	IC	10.71	10.33	10.27	10.03	10.73	10.12
	CT	10.86	12.56	11.34	12.35	11.16	11.62
DM	EEA	0.0025	0.0026	0.0030	0.0032	0.0035	0.0037
	ET	3.56	3.74	4.18	4.51	4.97	5.30
	CT	2.12	2.34	2.11	2.18	2.03	2.03

Remark: EEA denotes estimation error of Euler angle (L2-norm, expressed in radians); ET denotes estimation error of translation (L2-norm); IC denotes iteration count; CT denotes computation time (expressed in seconds). Each cell reports the average over all trials.

**Table 4:** Camera pose estimation results obtained by different methods.

	$\mathrm{Euler}\;\mathrm{Angle}/[10^{-5}$ rad]	Displacement/[mm]	Mean CCP Reprojection Error/[mm]	Epipolar Constraint Error RMS/[pix]
EPNP	[91.09, −1628.02, −7701.98	[315.96, −7.48, 77.16]	20.79	0.770
DQ-LS	[54.38, −9.426, −6792.29]	[271.49, 19.70, 58.32]	10.13	0.943
DLM-MSR	[174.278, −69.579, −6781.41]	[271.24, 20.04, 62.71]	10.24	0.944
DLM-SR	[4515.932, −0.606, −6808.39]	[310.26, −63.43, −5777.59]	5835.96	6.29
DM	[222.435, −69.184, −6781.95]	[271.26, 20.13, 62.70]	10.24	0.944
Design Value	[0, 0, 0]	[280, 0, 0]		

Remark: The CCP reprojection error is computed using the L2 norm.

**Table 5:** Pose estimation results.

Algorithm	Euler Angles (Yaw, Pitch, Roll)/Degree	Translation/mm
DLM-MSR	(−2.309, 0.140, −3.514) (3.131, −0.272, −4.981)	(21.82, 120.50, 258.43) (−1.01, 117.72, −373.73)
DM	(−2.309, 0.143, −3.514) (3.131, −0.272, −4.981)	(21.82, 120.50, 258.43) (−1.01, 117.72, −373.73)
DQ-LS	(−2.486, 4.345, −3.530) (3.288, −4.677, −5.113)	(32.15, 121.58, 318.20) (15.97, 117.78, −410.87)
EPNP	(1.769, −11.624, 3.805) (−14.490, −22.829, 40.962)	(888.51, 96.96, −312.61) (1370.40, 1412.53, 1317.17)
Design value	(0, 0, −4.89) (0, 0, −4.95)	

Remark: The top line in each cell corresponds to the 29/30 camera pair; the second line to the 30/31 pair.

**Table 6:** Comparison of re-projection errors.

Algorithm	Laser Point Sets 1/4 Projection Error		Laser Point Sets 2/3 Projection Error	
	MAE/[mm]	RMS/[mm]	MAE/[mm]	RMS/[mm]
DLM-MSR	(36.46, 19.85)	(36.50, 19.92)	(19.91, 8.03)	(19.92, 8.10)
DM	(37.36, 19.87)	(37.40, 19.94)	(20.98, 8.17)	(20.99, 8.18)

Remark: In each cell the first value corresponds to the 29/30 camera pair; the second to the 30/31 pair.

**Table 7:** Camera and laser parameters.

	Intrinsic Parameters $\left(k_{x},k_{y},u_{0},v_{0},k_{1},k_{2}\right)$	Laser Plücker Coordinates	Camera Pose (Euler Angle and Translation)
Camera 1	$\left(\begin{array}{c} 21,907.45,21,862.22,\\ 1242.68,1042.23,\\ 0.090,1.028 \end{array}\right)$	$L_{11}=\left(\begin{array}{c} 0.017,-0.022,0.99,\\ -80.39,216.73,6.38 \end{array}\right)$ $L_{12}=\left(\begin{array}{c} -0.0029,-0.030,0.99,\\ -75.70,-162.56,-5.18 \end{array}\right)$ $L_{13}=\left(\begin{array}{c} 0.029,0.017,0.99,\\ 76.68,201.32,-7.99 \end{array}\right)$ $L_{14}=\left(\begin{array}{c} -0.036,0.021,0.99,\\ 80.27,-178.50,6.47 \end{array}\right)$	$\theta =\left[\begin{array}{c} 0.0465\\ -0.0209\\ -0.0056 \end{array}\right]$ $t=\left[\begin{array}{c} -1.66\\ -159.90\\ -1.43 \end{array}\right]$
Camera 2	$\left(\begin{array}{c} 21,964.88,21,919.94,\\ 1242.97,1009.57,\\ 0.0150,0.606 \end{array}\right)$	$L_{21}=\left(\begin{array}{c} 0.0019,-0.018,0.99,\\ -83.21,202.03,5.07 \end{array}\right)$ $L_{22}=\left(\begin{array}{c} -0.051,-0.069,0.99,\\ -80.50,-177.54,-7.80 \end{array}\right)$ $L_{23}=\left(\begin{array}{c} 0.0030,0.027,0.99,\\ 79.42,215.73,-6.19 \end{array}\right)$ $L_{24}=\left(\begin{array}{c} 0.0027,0.020,0.99,\\ 85.21,-165.07,3.11 \end{array}\right)$	

## Data Availability

Dataset available on request from the authors.

## Abbreviations

The following abbreviations are used in this manuscript:

RTL	Road Tunnel Lining
RTL-D	RTL Deformation
RTL-ADs	RTL Appearance Defects
RTL-IDs	RTL Internal Defects
RIC	Road Tunnel Lining Inspection Car
FOVs	Fields of View
ASC	Area-scanning Camera
LSC	Line-scanning Camera
LSL	Line-scanning Laser
SVD	Singular Value Decomposition
NCC	Normalized Cross-correlation
PNP	Perspective-n-Point
NCCP	Non-corresponding Control Point
CCP	Corresponding Control Point
DQ	Dual Quaternions
MCC	Maximum Correntropy Criterion
DLM	DQ Laplace–MCC algorithm
RMS	Root Mean Square
MAE	Mean Absolute Error
MSR	Modified Silverman’s Rule
EEA	Estimation Error of Euler Angle
ET	Estimation Error of Translate
CT	Computation Time
ICE	Windows Image Composite Editor

## Appendix A

**Proof** **of** **Theorem** **1.** **Case** **1**: When only one line is involved in the calculation, from Equation (35a), we have the following:

(A1) $$\Gamma =\left[\begin{array}{cc} 0 & -{\left(L^{\prime }-L\right)}^{T}\\ L^{\prime }-L & {\left[L^{\prime }+L\right]}_{\times } \end{array}\right]=\left[\begin{array}{cc} 0 & -a^{T}\\ a & {\left[b\right]}_{\times } \end{array}\right]$$

where $a^{T}=\left[\begin{array}{ccc} a_{1} & a_{2} & a_{3} \end{array}\right]$, $b^{T}=\left[\begin{array}{ccc} b_{1} & b_{2} & b_{3} \end{array}\right],a_{i}=L_{i}^{\prime }-L_{i}$, and $b_{i}=L_{i}^{\prime }+L_{i}$. It is well known that the rank of a 3×3 skew-symmetric matrix is 2. Therefore, when the rotation matrix $R=I_{3}$, we have $\mathrm{rank}\left(\Gamma \right)=\mathrm{rank}\left({\left[b\right]}_{\times }\right)=2$; and not all $a_{i}$ are 0 if $R\neq I_{3}$. Assume $a_{1}\neq 0$, and after elementary row transformations, Γ becomes the following:

(A2) $$\Gamma \to \left[\begin{array}{cccc} a_{1} & 0 & -b_{3} & b_{2}\\ 0 & 1 & a_{2}/a_{1} & a_{3}/a_{1}\\ 0 & 0 & \gamma & 0\\ 0 & 0 & 0 & \gamma \end{array}\right]$$

where $\gamma =b_{1}+a_{3}/a_{1}b_{3}+a_{2}/a_{1}b_{2}$. Substituting $a_{i}=L_{i}^{\prime }-L_{i}$ and $b_{i}=L_{i}^{\prime }+L_{i}$ into Equation (A2), we obtain the following:

(A3) $$\gamma =\frac{{\left\|L^{\prime }\right\|}_{2}^{2}-{\left\|L\right\|}_{2}^{2}}{L_{1}^{\prime }-L_{1}}=0\left(∵{\left\|L^{\prime }\right\|}_{2}={\left\|L\right\|}_{2}\right)$$

Therefore, if only one line involved in the calculation is parallel, $\mathrm{rank}\left(\Gamma \right)=2$.

**Case 2**: When more than two lines are involved in the calculation, and they are all parallel, based on the properties of the matrix rank, we have $\mathrm{rank}\left(\Gamma \right)=2$.

**Case 3**: When two or more lines with different directions are involved, considering the case where $R=I_{3}$, it is clear that $\mathrm{rank}\left(\Gamma \right)\equiv 3$.

**Case 4**: When two or more lines with different directions are involved and $R\neq I_{3}$, assuming $a_{i1}\neq 0$ and $a_{j1}\neq 0$, we have the following:

(A4) $$\Gamma \to \left[\begin{array}{c} \begin{array}{cccc} 1 & 0 & -b_{i3}/a_{i1} & b_{i2}/a_{i1}\\ 0 & 1 & a_{i2}/a_{i1} & a_{i3}/a_{i1}\\ 1 & 0 & -b_{j3}/a_{j1} & b_{j2}/a_{j1}\\ 0 & 1 & a_{j2}/a_{j1} & a_{j3}/a_{j1} \end{array}\\ 0_{4\times 4} \end{array}\right]\to \left[\begin{array}{c} \begin{array}{cccc} 1 & 0 & -b_{i3}/a_{i1} & b_{i2}/a_{i1}\\ 0 & 1 & a_{i2}/a_{i1} & a_{i3}/a_{i1}\\ 0 & 0 & \psi_{1} & \psi_{2}\\ 0 & 0 & \phi_{1} & \phi_{2} \end{array}\\ 0_{4\times 4} \end{array}\right]$$

where $\psi_{1}=-b_{j3}/a_{j1}+b_{i3}/a_{i1}$, $\psi_{2}=b_{j2}/a_{j1}-b_{i2}/a_{i1}$, $\phi_{1}=a_{j2}/a_{j1}-a_{i2}/a_{i1}$, and $\phi_{2}=a_{j3}/a_{j1}-a_{i3}/a_{i1}$. Because the lines involved are not parallel, $\phi_{1}$ and $\phi_{2}$ cannot both be zero. Setting $\psi_{1}=0$ and $\psi_{2}=0$, we have $a_{i1}/a_{j1}=b_{i2}/b_{j2}=b_{i3}/b_{j3}=\lambda$, and expanding it obtain the following:

(A5) $$\begin{array}{c} \left({\bar{r}}_{1}-e_{1}^{T}\right)\left(L_{i}-\lambda L_{j}\right)=0\\ \left({\bar{r}}_{2}+e_{2}^{T}\right)\left(L_{i}-\lambda L_{j}\right)=0\\ \left({\bar{r}}_{3}+e_{3}^{T}\right)\left(L_{i}-\lambda L_{j}\right)=0 \end{array}$$

where ${\bar{r}}_{i}$ is the i-th row element of R, and $e_{i}={\left[\begin{array}{ccc} \delta_{i1} & \delta_{i2} & \delta_{i2} \end{array}\right]}^{T}$, with the following:

(A6) $$\delta_{ij}=\left\{\begin{array}{c} 1,\;\mathrm{if}\;i=j\\ 0,\;\mathrm{if}\;i\neq j \end{array}\right.$$

Equation (A5) can be written as follows:

(A7) $$R_{e}L_{i}=\lambda R_{e}L_{j}$$

where $R_{e}=R-\mathrm{diag}\left(\left[-1,\;1,\;1\right]\right)$. Clearly, if $R\left(0,0\right)\neq 1$ then the Euler angle about the X-axis is not 0, then $R_{e}$ is invertible, which implies $L_{i}=\lambda L_{j}$, meaning $L_{i}$ and $L_{j}$ are parallel, contradicting the assumption. If the Euler angle about the X-axis is 0, it must hold that $a_{i1}=0$ and $a_{j1}=0$, which contradicts the assumption. Therefore, $\psi_{1}$ and $\psi_{2}$ cannot both be 0, and thus $\mathrm{rank}\left(\Gamma \right)\geq 3$.

To prove that $\mathrm{rank}\left(\Gamma \right)\equiv 3$, we need to show that $\det\left(\left[\begin{array}{cc} \psi & \phi \end{array}\right]\right)\equiv 0$, that is ψ=Aϕ. Given $a_{i}=L_{i}^{\prime }-L_{i}=\left(R-I\right)L_{i}$ and $b_{i}=L_{i}^{\prime }+L_{i}=\left(R+I\right)L_{i}$, we have $b_{i}=Ma_{i}$, $b_{j}=Ma_{j}$, where $M=\left(R+I\right){\left(R-I\right)}^{-1}={\left[\begin{array}{ccc} {\bar{m}}_{1}^{T} & {\bar{m}}_{2}^{T} & {\bar{m}}_{3}^{T} \end{array}\right]}^{T}$, with $R\neq I_{3}$. Thus we obtain the following:

(A8) $$\begin{array}{ll} \psi & =\left[\begin{array}{c} -\frac{b_{j3}}{a_{j1}}+\frac{b_{i3}}{a_{i1}}\\ \frac{b_{j2}}{a_{j1}}-\frac{b_{i2}}{a_{i1}} \end{array}\right]=\frac{1}{a_{i1}a_{j1}}\left[\begin{array}{c} -a_{i1}b_{j3}+b_{i3}a_{j1}\\ a_{i1}b_{j2}-b_{i2}a_{j1} \end{array}\right]\\ & =\frac{1}{a_{i1}a_{j1}}\left[\begin{array}{c} -a_{i1}{\bar{m}}_{3}a_{j}+{\bar{m}}_{3}a_{i}a_{j1}\\ a_{i1}{\bar{m}}_{2}a_{j}-{\bar{m}}_{2}a_{i}a_{j1} \end{array}\right]\\ & =\frac{1}{a_{i1}a_{j1}}\left[\begin{array}{cc} \begin{array}{c} -m_{32} \end{array} & m_{22}\\ -m_{33} & m_{23} \end{array}\right]\left[\begin{array}{c} a_{i1}a_{j2}-a_{i2}a_{j1}\\ a_{i1}a_{j3}-a_{i3}a_{j1} \end{array}\right]\\ & =A\phi \end{array}$$

Therefore, under the conditions of the problem, there is $\mathrm{rank}\left(\Gamma \right)\equiv 3$. Because $L_{i}$ and $L_{j}$ are unit vectors, their elements cannot all be zero, and it can be verified that when $a_{im}\neq 0$ and $a_{jn}\neq 0$, we still have $\mathrm{rank}\left(\Gamma \right)\equiv 3$. □
